# Umbrella Review on Associations Between Single Nucleotide Polymorphisms and Lung Cancer Risk

**DOI:** 10.3389/fmolb.2021.687105

**Published:** 2021-09-03

**Authors:** Xiaoying Li, Qijun Wu, Baosen Zhou, Yashu Liu, Jiale Lv, Qing Chang, Yuhong Zhao

**Affiliations:** ^1^ Department of Clinical Epidemiology, Shengjing Hospital of China Medical University, Shenyang, China; ^2^ Clinical Research Center, Shengjing Hospital of China Medical University, Shenyang, China; ^3^ Department of Clinical Epidemiology, First Affiliated Hospital of China Medical University, Shenyang, China

**Keywords:** umbrella review, lung cancer, risk, single nucleotide polymorphism, association

## Abstract

The aim is to comprehensively and accurately assess potential relationships between single nucleotide polymorphisms (SNP) and lung cancer (LC) risk by summarizing the evidence in systematic reviews and meta-analyses. This umbrella review was registered with the PROSPERO international prospective register of systematic reviews under registration number CRD42020204685. The PubMed, Web of Science, and Embase databases were searched to identify eligible systematic reviews and meta-analyses from inception to August 14, 2020. The evaluation of cumulative evidence was conducted for associations with nominally statistical significance based on the Venice criteria and false positive report probability (FPRP). This umbrella review finally included 120 articles of a total of 190 SNP. The median number of studies and sample size included in the meta-analyses were five (range, 3–52) and 4 389 (range, 354–256 490), respectively. A total of 85 SNP (in 218 genetic models) were nominally statistically associated with LC risk. Based on the Venice criteria and FPRP, 13 SNP (in 22 genetic models), 47 SNP (in 99 genetic models), and 55 SNP (in 94 genetic models) had strong, moderate, and weak cumulative evidence of associations with LC risk, respectively. In conclusion, this umbrella review indicated that only 13 SNP (of 11 genes and one miRNA) were strongly correlated to LC risk. These findings can serve as a general and helpful reference for further genetic studies.

## Introduction

Lung cancer (LC) is associated with high morbidity and mortality rates and, thus, remains a serious threat to human health (Torre et al., 2015; Siegel et al., 2020). LC is generally discovered at advanced stages due to inconspicuous symptoms at the early stage of disease and the lack of effective and convenient screening methods (Nasim et al., 2019). Therefore, risk factors and biomarkers of the carcinogenesis and progression of LC should be explored for application in screening and clinical practice. Although smoking is a major risk factor, some LC patients have no history of smoking, indicating that other factors, such as second-hand smoke, indoor air pollution, and genetic factors, can promote the onset and progression of LC (Rivera and Wakelee, 2016).

Molecular epidemiological and experimental studies have shown that genetic variations play important roles in the occurrence of LC (Malhotra et al., 2016). A single nucleotide polymorphism (SNP), which is defined as a nucleotide variation with a frequency of greater than 1% in a population, is the most common form of genetic variation in the human genome. A growing number of studies on relationships between SNP and LC risk have been published in recent years. Systematic reviews and meta-analyses with relatively high levels of epidemiological evidence have summarized the associations between a SNP (or certain SNP) and LC risk, because the results have been somewhat inconsistent (Lau et al., 1998). However, the associations identified by systematic reviews and meta-analyses might be not accurate owing to the influence of various factors, such as publication bias (Ioannidis, 2005).

Dong et al. evaluated the results of meta-analyses and pooled analyses along with the false positive report probability (FPRP) to summarize the genetic susceptibility to cancer and found only 11 significant associations between genetic variations and LC risk (Dong et al., 2008). Marshall et al. mainly used the results of meta-analyses to review genetic susceptibility to LC which was identified with a candidate gene approach (Marshall and Christiani, 2013). In 2017, Liu et al. utilized the Venice criteria and FPRP to evaluate the results of meta-analyses to further summarize genetic associations with the risk of LC and found only 15 SNP with strong evidence (Liu et al., 2017). However, to the best of our knowledge, an umbrella review that extracts data, rather than the results, of systematic reviews and meta-analyses to calculate and evaluate the associations between SNP and LC risk has not been reported at present. Therefore, in order to comprehensively and accurately assess the relationships between SNP and LC risk, the present umbrella review was conducted based on the Venice criteria and FPRP.

## Methods

We conducted an umbrella review, which systematically collected and evaluated systematic reviews and meta-analyses of a specific research topic (Ioannidis, 2009). The umbrella review followed the PRISMA (Preferred Reporting Items for Reviews and Meta-analysis) and MOOSE (Meta-analyses of Observational Studies in Epidemiology) guidelines (Stroup et al., 2000; Moher et al., 2009). This umbrella review was registered with the PROSPERO 2020 international prospective register of systematic reviews under the registration number CRD42020204685.

### Literature Search

Eligible systematic reviews and meta-analyses published until August 14, 2020 were retrieved from the PubMed, Web of Science, and Embase databases with a combination of subject headings and free terms, as detailed in Supplementary Additional file S1. In addition, references of eligible articles were searched to avoid omissions.

### Eligibility Criteria

The inclusion criteria for article eligibility were: 1) systematic reviews or meta-analyses with quantitative synthesis; 2) investigations of the association between SNP and LC risk; 3) inclusion limited to observational studies, while excluding cross-sectional studies; 4) case-control studies or genome-wide association studies (GWAS) included in the meta-analyses that provided the number of cases and controls, and cohort studies included in the meta-analyses that provided the number of cases and population participants; 5) providing the genotyping data or specific relative risk estimates (risk ratio, odds ratio) with the 95% confidence interval (CI) of each included study; 6) included at least three studies; and 7) the article was written in English.

The exclusion criteria of eligible articles were: 1) included studies whose subjects were non-human, or studies without cancer-free controls; 2) included family-based studies; 3) investigations of variants with ranges greater than one SNP; 4) evaluation of the diagnosis, survival, or recurrence of LC; 5) meta-analyses or systematic reviews based on individual data; and 6) unpublished articles, published articles in abstracts only, letters to editors, and editorial comments.

If there was more than one eligible meta-analysis of the same SNP, the most recently published one (the time was subject to the deadline for including literature in the meta-analyses) with the corresponding data described in inclusion criteria 4) and 5) were retained because the most recent meta-analysis usually had the largest sample size (although sometimes smaller because of the stricter inclusion criteria) (Dong et al., 2008). If an article conducted meta-analyses of more than one SNP individually, each was assessed separately. This umbrella review was intended to include as many ethnicities as possible. Thus, the vast majority of meta-analyses included two or more ethnicities, unless a SNP was only performed meta-analyses for single ethnicity. For SNP that were ultimately rated as “strong” by evaluation of cumulative evidence, sensitivity analysis was conducted. Eligible articles were searched by two investigators individually and a dedicated investigator was responsible for quality control and decisions on inconsistencies.

### Data Extraction

Two investigators separately extracted data from the eligible systematic reviews and meta-analyses and a dedicated investigator conducted quality control and resolved inconsistencies. For each eligible article, the extracted data included 1) the name of the first author, 2) year of publication, 3) examined SNP, 4) gene name, 5) the number of included studies, 6) genotyping data or specific relative risk estimates (risk ratio, odds ratio) with the 95% CI for each of the included studies (genotyping data was preferred), 7) epidemiological design (case-control study, GWAS, or cohort study) of each study, 8) the number of cases and controls (for case-control studies and GWAS) or the number of cases and population participants (for cohort studies) of each study, and 9) the probability (*p*) value of the Hardy-Weinberg equilibrium (HWE) test for each of the included studies.

### Quality Assessment of Included Articles

Two investigators separately used the AMSTAR tool to evaluate the quality of the included articles and a third investigator was responsible for quality control and resolving inconsistencies (Shea et al., 2007). The AMSTAR tool includes 11 criterion items that are scored as 1 point for a positive or 0 points for other answers. The total score is the sum of the 11 items as follows: ≥8 points was considered as high quality; 4–7 points as moderate quality; and ≤3 points as low quality (Neuenschwander et al., 2019).

### Statistical Analysis

If the HWE results of the controls were not available, the HWE was evaluated with the chi square test. As there is no consensus on an optimal genetic model for the study of SNP, five commonly used genetic models were used for analysis, unless the corresponding data for some genetic models were not available. The five commonly used genetic models included the heterozygote comparison model (model 1), the homozygote comparison model (model 2), the dominant model (model 3), the recessive model (model 4), and the allele model (model 5) (i.e., if a SNP is 1/2, the heterozygote comparison model: 12 vs 11; the homozygote comparison model: 22 vs 11; the dominant model: 12 + 22 vs 11; the recessive model: 22 vs 11 + 12; the allele model: 2 vs 1).

### Assessment of Pooled Effects and Heterogeneity

Fixed-effects and random-effects models were used to calculate the pooled effects with 95% CI for each meta-analysis (DerSimonian and Laird, 1986; Lau et al., 1997). For the sake of conservativeness, the main inferences were based on a random-effects model and *p* < 0.05 (random-effects model) was considered nominally statistically significant for each meta-analysis (Vineis et al., 2009). The 95% prediction intervals of the summary effect estimates (random-effects model) were further evaluated to account for the heterogeneity between studies and suggest the uncertainty of an effect that would be expected in a new study exploring the same relationship (Higgins et al., 2009; Riley et al., 2011). Between-study heterogeneity was assessed with the Cochran *Q* statistic and the *I* ^2^ statistic (Higgins and Thompson, 2002). For the Cochran *Q* statistic, *p* < 0.10 was considered statistically significant (Lau et al., 1997). *I* ^2^ > 50% is often considered to indicate a large degree of heterogeneity. The 95% CI of *I* ^2^ was calculated based on the method described by Ioannidis et al. (Ioannidis et al., 2007).

### Evaluation of Bias

For SNP with nominal statistical significance, four methods were used to assess bias. First, for nominally statistically significant relationships, we examined whether the relationships were lost by excluding the first published studies (Vineis et al., 2009). Second, for nominally statistically significant relationships, we also assessed whether the associations were lost by excluding studies that violated the HWE (*p* < 0.05) (Trikalinos et al., 2006). Third, assessment of the small-study effect was conducted to determine whether relatively small studies, as compared to relatively large studies, were apt to give higher risk estimates. The asymmetry test, as described by Egger et al. (1997), was used to assess the small-study effect, which was considered to exist when: 1) the *p*-value of the Egger’s test was <0.10 and 2) the larger studies had a more conservative effect size than the random-effects meta-analysis (Carvalho et al., 2016). Fourth, assessment of excess significance was performed using the Ioannidis test (Ioannidis and Trikalinos, 2007). Briefly, evaluation of excess significance was to compare the observed number of studies of nominally significant results (O) with the expected number of significant results (E). Excess significance was considered to exist when the *p*-value of the Ioannidis test was <0.10 and O > E. All analyses were two-sided and performed with Stata 11 software (Stata LLC, College Station, TX, United States).

### Evaluation of Cumulative Evidence

The cumulative evidence of SNP with nominal statistical significance was further evaluated. First, the strength of the evidence, as an indicator of epidemiological credibility, was evaluated using the Venice criteria (Ioannidis et al., 2008) that have been applied in previous studies (Vineis et al., 2009; Giannakou et al., 2018; Yang et al., 2019). The grading criteria included three items (amount of evidence, replication, and protection from bias), which were rated as A, B, or C, as described in detail in Table 1. If the sample size of the rarer allele in a meta-analysis could not be directly obtained, the value was calculated based on the minor allele frequency (MAF) retrieved from the SNP database of the National Center for Biotechnology Information (https://www.ncbi.nlm.nih.gov/snp/). MAF usually refers to the frequency of alleles that are uncommon in a given population. Finally, an association with a rating of AAA was considered strong, while a rating of C for any of the three items was considered weak. All other ratings were considered moderate.

**TABLE 1:** Criteria for evaluation of epidemiological credibility.

Criteria	Categories
Amount of evidence	A: The sample size for the rarer genotype/allele in a meta-analysis is greater than 1000
—	B: The sample size for the rarer genotype/allele in a meta-analysis is 100–1000
—	C: The sample size for the rarer genotype/allele in a meta-analysis is less than 100
Replication	A: *I* ^2^ < 25 and 95% *PI* excluding the null value
—	B: *I* ^2^ < 25 and 95% *PI* including the null value, or *I* ^2^ 25–50%
—	C: *I* ^2^ > 50% or no nominally statistically significant association in a meta-analysis
Protection from bias	A: The summary effect size (in the random-effects model) is greater than 1.15 or less than 0.87, and the following four situations do not occur: 1) nominal statistical significance is lost with the exclusion of the first published study; 2) nominal statistical significance is lost with the exclusion of studies where HWE is violated; 3) small study effects; 4) excess significance bias
—	B: there is no evidence of bias that would invalidate an association, but important information is missing
—	C: the summary effect size (in the random-effects model) is 0.87–1.15, or at least one of the following four situations occurs: 1) nominal statistical significance is lost with the exclusion of the first published study; 2) nominal statistical significance is lost with the exclusion of studies where HWE is violated; 3) small study effects; 4) excess significance bias

The FPRP is a Bayesian prophylactic against false reports of significant associations. The FPRP was calculated with the Excel spreadsheet on the Wacholder website (Wacholder et al., 2004). For FPRP calculations, the prior probability was preset to 0.05, the FPRP noteworthiness value was 0.2, and the statistical power of detecting an OR of 1.5 (for SNP with an increased risk) or an OR of 0.67 (for SNP with a decreased risk) was used, as described by Wacholder et al. (2004). If the FPRP value was less than 0.2, the association was considered noteworthy, as the association might be true. The strength of FPRP was divided into the following three categories: FPRP <0.05, strong; 0.05 < FPRP <0.2, moderate; and FPRP >0.2, weak. In order to more accurately evaluate the cumulative evidence, the Venice criteria and FPRP were combined. If the FPRP was rated as strong, the evidence strength determined with the Venice criteria was upgraded from moderate to strong or from weak to moderate. Otherwise, if the FPRP was rated as weak, the evidence strength determined with the Venice criteria was downgraded from strong to moderate or from moderate to weak (Liu et al., 2017).

## Results

### Overall Characteristics

**FIGURE 1:**
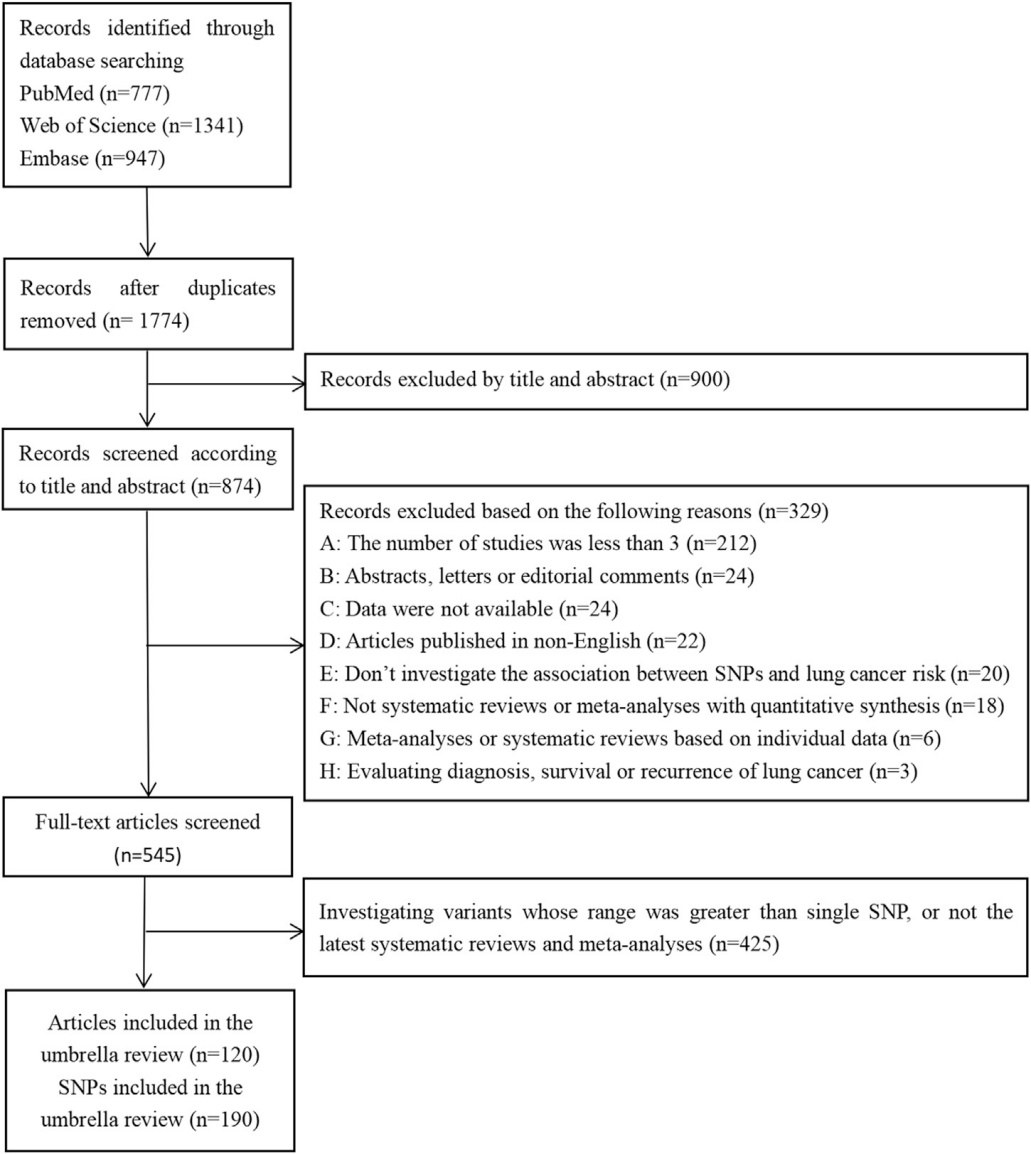
The screening process of records.

Of the 3,065 records initially retrieved from the PubMed, Web of Science, and Embase databases, 1,774 (57.88%) were retained after removing duplicates. Finally, 120 articles were included in the umbrella review (Figure 1), which referred to a total of 190 SNP.

The basic characteristics of the included articles are summarized in Supplementary Additional file S2. The majority of the included articles (*n* = 89, 74.17%) were published since 2015 (range, 2008–2020). With respect to the epidemiological design, most of the meta-analyses (91.58%) were synthesized by case-control studies as well as nested case-control studies. Some meta-analyses (8.42%) also included the training set and validated set of GWAS. Included subjects were from many regions of the world, as shown in Supplementary Additional file S2 and Supplementary Additional file S3.

The quality of all included articles was assessed and the results were listed in Supplementary Additional file S2. Based on the AMSTAR score, 13 (10.83%) of the included articles were considered as high quality, 99 (82.50%) as moderate quality, and 8 (6.67%) as low quality.

### Single Nucleotide Polymorphisms With Nominal Statistical Significance in the Meta-analyses

A total of 85 SNP were nominally statistically associated with LC risk in at least one genetic model. Of these 85 SNP, 83 were located on 54 genes and two were located on two miRNAs. The median number of studies included in the meta-analyses was six (range, 3–52). The median number of cases, controls, and total sample size included in the meta-analyses were 3,173 (range, 402–55,353), 3,578 (range, 396–239 337), and 7,016 (range, 798–256 490), respectively (Supplementary Additional file S4).

**FIGURE 2:**
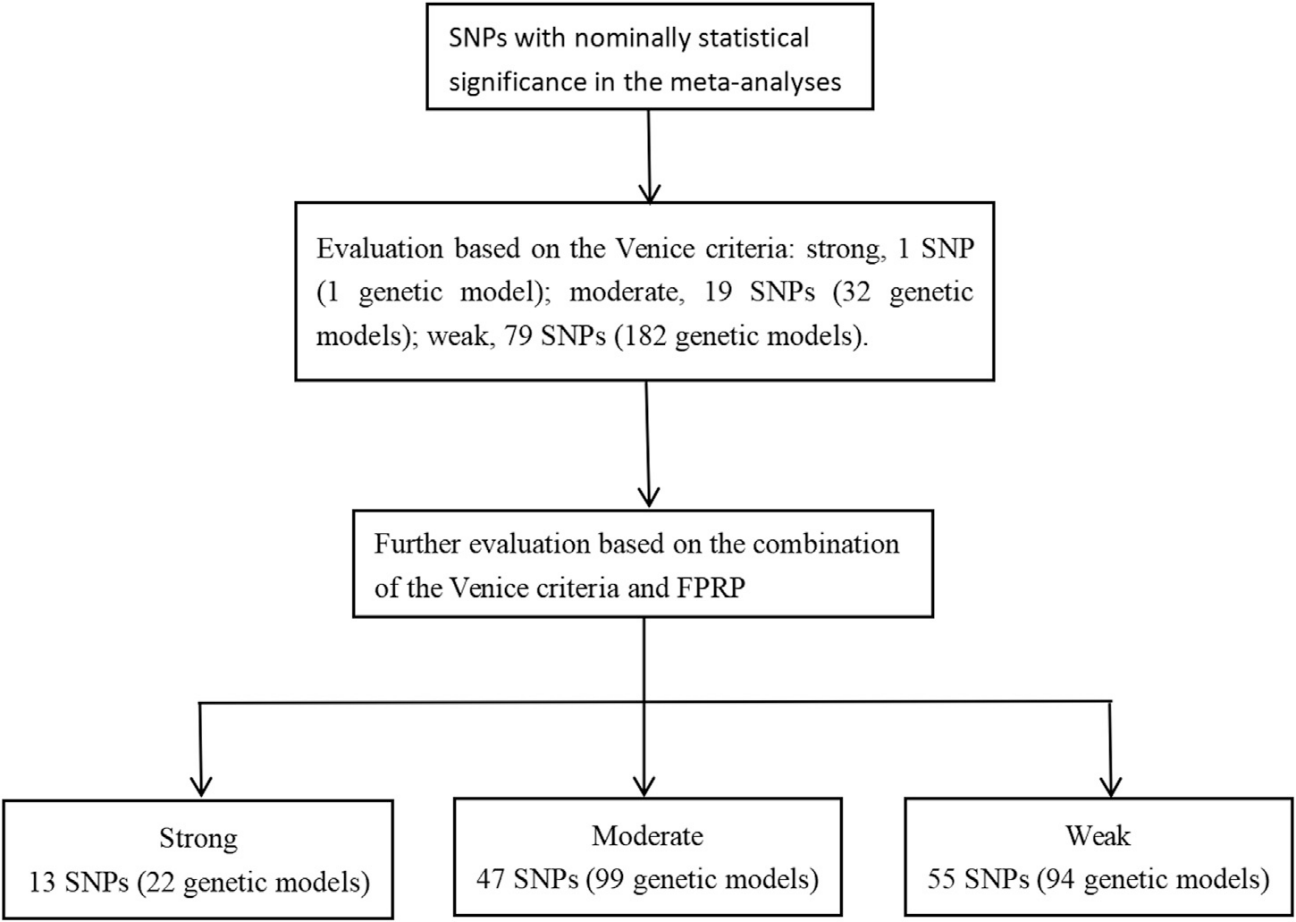
The evaluation process of cumulative evidence.

The Venice criteria were used to assess the strength of evidence (Figure 2 and Supplementary Additional file S5). Only rs31489 (model 1) of the *CLPTM1L* gene was rated as strong evidence. A total of 32 genetic models of 19 SNP and 182 genetic models of 79 SNP were rated as moderate and weak evidence, respectively.

The Venice criteria and FPRP were combined to more accurately evaluate the cumulative evidence (Figure 2, Table 2, Table 3, and Table 4). There were 22 genetic models of 13 SNP with strong cumulative evidence. These 13 SNP were located on 11 genes and one miRNA. Among these 13 SNP, rs664143, rs31489, rs4646903 rs1048943, rs2308321, rs2735383, rs2736098, rs1800975, rs3213245, and rs12740674 were associated with an increased risk of LC, while rs2240308, rs938682, and rs2031920 were associated with a decreased risk. There were 47 SNP with moderate cumulative evidence that referred to 99 genetic models. Of these 47 SNP, 34 referring to 78 genetic models were associated with an increased risk of LC, whereas 13 SNP referring to 21 genetic models were associated with a decreased risk. In addition, 94 genetic models of 55 SNP were rated as weak cumulative evidence. However, 3 genetic models of 3 SNP could not be graded according to the Venice criteria and, therefore, were not assigned a final rating because the sample size of the rarer genotype in the meta-analyses could not be obtained directly or calculated based on the MAF.

**TABLE 2:** Meta-analysis results of SNPs with the strong cumulative evidence based on the Venice criteria and FPRP.

SNPs	Gene name	Variant	Genetic modle	The number of studies	I^2^ (95%CI)	OR 95%CI (random effects)	*P* (R)	95%PI
rs664143	*ATM*	1G; 2A	1	4	0.0 (0, 85)	1.444 (1.181, 1.766)	<0.001	0.93, 2.25
rs2240308	*AXIN2*	1C; 2T	3	4	0.0 (0, 85)	0.703 (0.588, 0.840)	<0.001	0.48, 1.04
rs938682	*CHRNA3*	1T; 2C	5	6	48.0 (0, 79)	0.796 (0.724, 0.876)	<0.001	0.62, 1.03
rs31489	*CLPTM1L*	1A; 2C	2	10	29.7 (0, 66)	1.284 (1.166, 1.413)	<0.001	1.04, 1.59
—	—	—	3	10	0.0 (0, 62)	1.198 (1.123, 1.278)	<0.001	1.11, 1.29
rs4646903	*CYP1A1*	1C; 2T	2	41	35.1 (5, 56)	1.395 (1.161, 1.676)	<0.001	0.69, 2.82
—	—	—	5	41	41.1 (14, 59)	1.172 (1.085, 1.265)	<0.001	0.85, 1.62
rs1048943	*CYP1A1*	1 Ile; 2 Val	4	37	39.0 (9, 59)	1.626 (1.313, 2.013)	<0.001	0.73, 3.64
rs2031920	*CYP2E1*	1C; 2T	1	29	32.3 (0, 57)	0.796 (0.701, 0.904)	<0.001	0.53, 1.20
—	—	—	3	34	37.8 (6, 59)	0.801 (0.712.0.900)	<0.001	0.52, 1.23
rs2308321	*MGMT*	1 Ile; 2 Val	1	5	0.0 (0, 79)	1.198 (1.082, 1.326)	0.001	1.02, 1.41
—	—	—	3	5	8.9 (0, 81)	1.191 (1.063, 1.335)	0.003	0.95, 1.50
rs2735383	*NBS1*	1G; 2C	3	4	0.0 (0, 85)	1.187 (1.067, 1.321)	0.002	0.94, 1.50
—	—	—	4	4	10.0 (0, 86)	1.275 (1.109, 1.466)	0.001	0.89,1.83
rs2736098	*TERT*	1G; 2A	1	10	25.2 (0, 64)	1.199 (1.086, 1.323)	<0.001	0.97, 1.49
—	—	—	3	10	26.8 (0, 65)	1.305 (1.188, 1.434)	<0.001	1.06, 1.61
rs1800975	*XPA*	1G; 2A	4	16	12.6 (0, 50)	1.157 (1.056, 1.269)	0.002	0.97, 1.37
rs3213245	*XRCC1*	1T; 2C	2	7	0.0 (0, 71)	1.992 (1.422, 2.791)	<0.001	1.28, 3.10
—	—	—	4	7	0.0 (0, 71)	1.894 (1.365, 2.627)	<0.001	1.23, 2.91
rs12740674	miR-1262	1C; 2T	2	3	0.0 (0, 90)	1.738 (1.316, 2.295)	<0.001	0.29, 10.54
—	—	—	3	3	0.0 (0, 90)	1.209 (1.096, 1.333)	<0.001	0.64, 2.28
—	—	—	4	3	0.0 (0, 90)	1.667 (1.265, 2.199)	<0.001	0.28, 10.02

P(R): p value of the random effect.

**TABLE 3:** Cumulative evidence details of SNPs with the strong cumulative evidence based on the Venice criteria and FPRP.

SNPs	Genetic modle	*P* (Excluding the first published study)	*P* (Excluding studies with the violated HWE)	Small-study effect	Excess significance	Venice criteria	*P* (FPRP)	Cumulative evidence
rs664143	1	0.018	<0.001	No	No	Moderate (B/B/A)	0.010	Strong
rs2240308	3	0.001	<0.001	No	No	Moderate (B/B/A)	0.003	Strong
rs938682	5	0.004	<0.001	No	No	Moderate (A^a^/B/A)	<0.001	Strong
rs31489	2	0.005	<0.001	No	No	Moderate (A/B/A)	<0.001	Strong
—	3	0.003	<0.001	No	No	Strong (A/A/A)	<0.001	Strong
rs4646903	2	0.001	0.001	No	No	Moderate (A/B/A)	0.009	Strong
—	5	<0.001	<0.001	No	No	Moderate (A/B/A)	0.001	Strong
rs1048943	4	<0.001	0.001	No	No	Moderate (B/B/A)	0.001	Strong
rs2031920	1	<0.001	0.002	No	No	Moderate (B/B/A)	0.008	Strong
—	3	<0.001	NA	No	No	Moderate (B/B/B)	0.004	Strong
rs2308321	1	0.001	0.001	No	No	Moderate (B/A/A)	0.009	Strong
—	3	0.005	0.003	No	No	Moderate (B/B/A)	0.049	Strong
rs2735383	3	0.002	0.002	No	No	Moderate (A/B/A)	0.031	Strong
—	4	<0.001	0.001	No	No	Moderate (A/B/A)	0.012	Strong
rs2736098	1	0.001	<0.001	No	No	Moderate (A/B/A)	0.006	Strong
—	3	<0.001	<0.001	No	No	Moderate (A/B/A)	<0.001	Strong
rs1800975	4	0.003	0.015	No	No	Moderate (A/B/A)	0.036	Strong
rs3213245	2	<0.001	0.008	No	No	Moderate (B/A/A)	0.023	Strong
—	4	0.001	0.021	No	No	Moderate (B/A/A)	0.030	Strong
rs12740674	2	NA	<0.001	No	NO	Mderate (B/B/B)	0.012	Strong
—	3	NA	<0.001	No	No	Moderate (B/B/B)	0.003	Strong
—	4	NA	<0.001	No	No	Moderate (B/B/B)	0.024	Strong

NA: Not available;

a The sample size for the rarer allele in a meta-analysis was calculated based on the MAF offered by dbSNP of NCBI.

**TABLE 4:** Cumulative evidence details of SNPs with the moderate cumulative evidence based on the Venice criteria and FPRP.

SNPs	Gene name	Genetic modle	*P* (Excluding the first published study)	*P* (Excluding studies with the violated HWE)	Small-study effect	Excess significance	Venice criteria	*P* (FPRP)	Cumulative evidence
rs8034191	*AGPHD1*	1	<0.001	<0.001	No	Yes	Weak (A/A/C)	<0.001	Moderate
—	—	2	<0.001	<0.001	No	Yes	Weak (A/B/C)	<0.001	Moderate
—	—	3	<0.001	<0.001	No	Yes	Weak (A/B/C)	<0.001	Moderate
—	—	4	<0.001	<0.001	No	Yes	Weak (A/A/C)	<0.001	Moderate
—	—	5	<0.001	<0.001	No	Yes	Weak (A/B/C)	<0.001	Moderate
rs931794	*AGPHD1*	3	0.011	0.001	Yes	Yes	Weak (NA/B/C)	0.012	Moderate
rs1760944	*APEX1*	2	0.001	<0.001	No	Yes	Weak (A/A/C)	0.001	Moderate
—	—	4	0.007	0.001	No	Yes	Weak (A/B/C)	0.013	Moderate
—	—	5	0.001	<0.001	No	Yes	Weak (A/A/C)	0.002	Moderate
rs664143	*ATM*	2	0.005	0.001	No	Yes	Weak (B/B/C)	0.041	Moderate
—	—	3	0.006	<0.001	No	Yes	Weak (B/B/C)	0.006	Moderate
rs2240308	*AXIN2*	1	0.011	0.005	No	NO	Moderate (B/B/A)	0.087	Moderate
—	—	2	<0.001	<0.001	Yes	Yes	Weak (B/B/C)	0.007	Moderate
—	—	4	0.001	<0.001	No	Yes	Weak (B/B/C)	0.010	Moderate
—	—	5	<0.001	<0.001	Yes	Yes	Weak (A/A/C)	<0.001	Moderate
rs3117582	*BAT3*	5	<0.001	NA	No	Yes	Weak (A^a^/B/C)	<0.001	Moderate
rs6983267	*CASC8*	1	0.009	0.013	No	No	Moderate (A/B/A)	0.193	Moderate
—	—	2	<0.001	0.001	Yes	Yes	Weak (A/B/C)	0.011	Moderate
—	—	3	0.001	0.002	Yes	Yes	Weak (A/B/C)	0.032	Moderate
—	—	5	<0.001	0.001	Yes	Yes	Weak (A/B/C)	0.021	Moderate
rs151606	*CEP43*	5	NA	NA	No	NO	Weak (A/B/C)	0.018	Moderate
rs12212247	*CEP43*	5	NA	NA	Yes	Yes	Weak (A/A/C)	0.001	Moderate
rs1051730	*CHRNA3*	2	<0.001	<0.001	No	Yes	Weak (A/B/C)	<0.001	Moderate
—	—	3	<0.001	NA	No	Yes	Weak (A/C/C)	<0.001	Moderate
—	—	4	<0.001	<0.001	No	Yes	Weak (A/A/C)	<0.001	Moderate
—	—	5	<0.001	NA	No	Yes	Weak (A/B/C)	<0.001	Moderate
rs578776	*CHRNA3*	5	<0.001	<0.001	No	Yes	Weak (A^a^/A/C)	<0.001	Moderate
rs6495309	*CHRNA3*	1	0.001	<0.001	No	Yes	Weak (A/A/C)	<0.001	Moderate
—	—	2	<0.001	<0.001	No	Yes	Weak (A/C/C)	0.001	Moderate
—	—	3	<0.001	<0.001	No	Yes	Weak (A/B/C)	<0.001	Moderate
—	—	5	<0.001	<0.001	No	Yes	Weak (A/C/C)	0.001	Moderate
rs16969968	*CHRNA5*	1	<0.001	<0.001	No	Yes	Weak (A/B/C)	<0.001	Moderate
—	—	2	<0.001	<0.001	No	Yes	Weak (A/A/C)	<0.001	Moderate
—	—	3	<0.001	<0.001	No	Yes	Weak (A/B/C)	<0.001	Moderate
—	—	4	<0.001	<0.001	No	Yes	Weak (A/A/C)	<0.001	Moderate
—	—	5	<0.001	<0.001	No	Yes	Weak (A/B/C)	<0.001	Moderate
rs402710	*CLPTM1L*	5	<0.001	NA	No	Yes	Weak (A^a^/C/C)	<0.001	Moderate
rs401681	*CLPTM1L*	5	<0.001	NA	No	Yes	Weak (A^a^/A/C)	<0.001	Moderate
rs31489	*CLPTM1L*	1	0.008	<0.001	No	Yes	Weak (A/A/C)	0.005	Moderate
—	—	4	0.009	<0.001	No	No	Weak (A/C/C)	0.003	Moderate
—	—	5	0.007	<0.001	No	Yes	Weak (A/C/C)	<0.001	Moderate
rs2453176	*CNOT6*	5	NA	NA	No	Yes	Weak (A/B/C)	0.022	Moderate
rs231775	*CTLA-4*	5	0.546	<0.001	No	No	Weak (A/B/C)	0.008	Moderate
rs4646903	*CYP1A1*	1	0.004	0.004	No	No	Moderate (A/B/A)	0.052	Moderate
—	—	3	<0.001	NA	No	No	Weak (A/C/B)	0.002	Moderate
—	—	4	0.008	0.004	No	No	Moderate (A/B/A)	0.076	Moderate
rs1048943	*CYP1A1*	2	<0.001	0.001	No	No	Weak (B/C/A)	0.004	Moderate
—	—	5	0.001	0.002	No	No	Weak (A/C/A)	0.006	Moderate
rs1065852	*CYP2D6*	4	0.002	0.258	No	No	Weak (B/B/C)	0.017	Moderate
rs4646904	*CYP4F3*	5	NA	NA	No	Yes	Weak (A/B/C)	0.026	Moderate
rs12587742	*DCAF4*	5	NA	NA	No	Yes	Weak (A/B/C)	0.036	Moderate
rs2240980	*DCAF4*	5	NA	NA	No	Yes	Weak (A/A/C)	<0.001	Moderate
rs13181	*ERCC2*	1	<0.001	<0.001	Yes	No	Weak (A/B/C)	<0.001	Moderate
—	—	2	0.002	<0.001	Yes	No	Weak (A/B/C)	0.020	Moderate
—	—	3	<0.001	<0.001	Yes	No	Weak (A/B/C)	<0.001	Moderate
—	—	5	<0.001	<0.001	Yes	No	Weak (A/C/C)	<0.001	Moderate
rs11549467	*HIF-1α*	1	0.001	0.002	No	No	Weak (C/B/A)	0.046	Moderate
rs1800734	*hMLH1*	4	<0.001	0.065	No	Yes	Weak (B/B/C)	0.001	Moderate
rs2279744	*MDM2*	4	0.020	0.005	No	No	Moderate (A/B/A)	0.084	Moderate
rs2285053	*MMP2*	5	0.228	<0.001	No	No	Weak (A/B/C)	0.002	Moderate
rs11568818	*MMP7*	1	0.117	<0.001	No	Yes	Weak (C/B/C)	0.024	Moderate
—	—	3	0.148	<0.001	No	Yes	Weak (C/B/C)	0.016	Moderate
rs1801133	*MTHFR*	1	0.001	0.026	Yes	No	Weak (A/C/C)	0.021	Moderate
—	—	2	<0.001	0.003	Yes	No	Weak (A/C/C)	0.006	Moderate
—	—	3	<0.001	0.007	Yes	No	Weak (A/C/C)	0.006	Moderate
—	—	5	<0.001	0.005	Yes	No	Weak (A/C/C)	0.007	Moderate
rs2735383	*NBS1*	2	<0.001	<0.001	No	Yes	Weak (A/B/C)	0.001	Moderate
—	—	5	<0.001	<0.001	No	Yes	Weak (A/B/C)	0.001	Moderate
rs1553232011	*NEXN-AS1*	5	NA	NA	Yes	Yes	Weak (A/B/C)	<0.001	Moderate
rs2890658	*PD-L1*	1	<0.001	<0.001	Yes	Yes	Weak (C/B/C)	0.001	Moderate
—	—	3	<0.001	<0.001	Yes	YEes	Weak (C/B/C)	<0.001	Moderate
—	—	5	<0.001	<0.001	Yes	Yes	Weak (B/B/C)	<0.001	Moderate
rs1800624	*RAGE*	2	0.073	0.001	No	No	Weak (B/B/C)	0.049	Moderate
rs2853677	*TERT*	1	0.001	<0.001	No	Yes	Weak (B/B/C)	0.005	Moderate
—	—	2	<0.001	<0.001	No	Yes	Weak (B/A/C)	<0.001	Moderate
—	—	3	<0.001	<0.001	No	Yes	Weak (B/A/C)	<0.001	Moderate
—	—	4	0.001	<0.001	No	Yes	Weak (B/B/C)	0.006	Moderate
—	—	5	<0.001	<0.001	No	Yes	Weak (A/A/C)	<0.001	Moderate
rs2736100	*TERT*	1	<0.001	<0.001	No	Yes	Weak (A/B/C)	<0.001	Moderate
—	—	2	<0.001	<0.001	No	Yes	Weak (A/C/C)	<0.001	Moderate
—	—	3	<0.001	NA	No	Yes	Weak (A/C/C)	<0.001	Moderate
—	—	4	<0.001	<0.001	No	Yes	Weak (A/C/C)	<0.001	Moderate
—	—	5	<0.001	NA	No	Yes	Weak (A/C/C)	<0.001	Moderate
rs2853669	*TERT*	2	0.014	0.002	No	No	Moderate(B/B/A)	0.100	Moderate
—	—	4	0.015	<0.001	No	Yes	Weak(B/B/C)	0.005	Moderate
rs2736098	*TERT*	2	<0.001	<0.001	No	Yes	Weak (A/A/C)	<0.001	Moderate
—	—	4	<0.001	<0.001	No	Yes	Weak (A/A/C)	<0.001	Moderate
—	—	5	<0.001	<0.001	No	Yes	Weak (A/A/C)	<0.001	Moderate
rs2853676	*TERT*	5	0.005	0.005	No	No	Weak (A/B/C)	0.030	Moderate
rs1544410	*VDR*	5	<0.001	0.020	Yes	No	Weak (A/C/C)	0.021	Moderate
rs699947	*VEGF*	5	0.007	0.126	No	No	Weak (A/C/C)	0.048	Moderate
rs3213245	*XRCC1*	5	0.019	0.012	No	Yes	Weak (A/C/C)	0.038	Moderate
rs3769201	*ZAK*	5	NA	NA	Yes	No	Weak (A/A/C)	<0.001	Moderate
rs722864	*ZAK*	5	NA	NA	No	No	Weak (A/A/C)	<0.001	Moderate
rs12740674	miR-1262	1	NA	0.003	No	No	Moderate(B/B/B)	0.059	Moderate
—	—	5	NA	<0.001	No	Yes	Weak (A/B/C)	<0.001	Moderate
rs2910164	miR-146a	2	0.002	0.003	Yes	No	Weak (A/C/C)	0.046	Moderate
—	—	4	0.001	0.001	Yes	No	Weak (A/C/C)	0.009	Moderate
—	—	5	<0.001	0.001	Yes	No	Weak (A/C/C)	0.004	Moderate

NA: Not available;

a The sample size for the rarer allele in a meta-analysis was calculated based on the MAF offered by dbSNP of NCBI.

### Single Nucleotide Polymorphisms Without Nominal Statistical Significance in the Meta-analyses

A total of 148 SNP were not nominally statistically significant in at least one genetic model (Supplementary Additional file S6). Of these, 143 SNP were located on 83 genes, four were located on four miRNAs, and one was located on pre-miR-27a. The median number of studies included in the meta-analyses was five (range, 3–52). The median number of cases, controls, and total sample size included in the meta-analyses were 1,757 (range, 150–17,318), 2,063 (range, 204–35,755), and 3,858 (range, 354–39,445), respectively. Among these 148 SNP, 81 were not nominally statistically significant in any of the five genetic models.

### Sensitivity Analyses

Sensitivity analyses of the corresponding models of SNP with the strong cumulative evidence (Supplementary Additional file S7) failed to find that the results were influenced by any single study, in addition to rs2308321 (models 1 and 3).

## Discussion

Based on the Venice criteria and FPRP, 13 SNP had strong cumulative evidence of associations with LC risk in at least one genetic model, 47 SNP (in 99 genetic models) had moderate cumulative evidence, and 55 SNP (in 94 genetic models) had weak cumulative evidence. In general, the results of this umbrella review were not in very good agreement with the review by Liu et al. (2017) because the latter evaluated cumulative evidence of genetic polymorphisms and LC risk based on the existing meta-analysis results and relatively loose Venice criteria. The 13 SNP with strong cumulative evidence were located on 11 genes and one miRNA. Based on the predictions of the GSCALite website (http://bioinfo.life.hust.edu.cn/web/GSCALite/), eight (72.7%) of these 11 genes (*AXIN2*, *CHRNA3*, *CLPTM1L*, *CYP1A1*, *MGMT*, *NBS1*, *TERT*, *XPA*) might be involved in one or more pathways related to LC (Liu et al., 2018).

The *AXIN2* (axis inhibition protein 2) gene, also known as *AXIL* and *ODCRCS*, is a negative regulator of the Wnt/β-catenin signaling pathway that may play an important role in tumorigenesis (Kikuchi, 1999; Hughes and Brady, 2005). SNP rs2240308 is located in the *AXIN2* coding region (exon1) at 17q24.1, suggesting that rs2240308 may significantly influence *AXIN2* gene expression. In the present umbrella review, rs2240308 was associated with susceptibility to LC, as suggested by the strong cumulative evidence in the dominant model. As compared with the CC genotype, the TT + CT genotype was associated with a significantly reduced risk of LC. However, the sample size of rs2240308 in the meta-analysis was relatively small, thus further investigations are necessary.

The *CHRNA3* (cholinergic receptor nicotinic α3) gene, also known as *LNCR2*, *PAOD2*, *BAIPRCK*, and *NACHRA3*, encodes the α3 nAChR (nicotinic acetylcholine receptor) subunit. A study by Paliwal et al. demonstrated that depletion and restoration of *CHRNA3* expression induces and resists cell apoptosis, respectively (Paliwal et al., 2010). Moreover, Egleton et al. indicated that activation of nAChRs may act as tumor promoters to stimulate the development of LC cells and suppress apoptosis (Egleton et al., 2008). The SNP rs938682 of the *CHRNA3* gene is located at 15q25.1. In the current umbrella review, rs938682 of the *CHRNA3* gene had a strong cumulative evidence for the association with LC risk in the allele model. In contrast to the T allele, the C allele was associated with a reduced risk of LC.

The *CLPTM1L* (cleft lip and palate transmembrane protein 1) gene, also known as *CRR9*, is a LC risk candidate gene that was found to be overexpressed in human lung tumor cell lines and lung tumors (James et al., 2012; Ni et al., 2012). SNP rs31489 of the *CLPTM1L* gene is located at 5p15.3. In this umbrella review, SNP rs31489 was strongly associated with susceptibility to LC in the homozygote comparison model and the dominant model. As compared to the AA genotype, the CC and CC + AC genotypes were associated with an increased risk of LC.

The *CYP1A1* (cytochrome P450 family 1 subfamily A member 1) gene, also known as *AHH*, *AHRR*, *CP11*, *CYP1*, *CYPIA1*, *P1-450*, *P450-C*, and *P450DX*, encodes a phase I enzyme that adjusts the metabolic activation of important tobacco procarcinogens, such as polycyclic aromatic hydrocarbons and aromatic amines, and might influence susceptibility to LC by regulating the metabolism of environmental carcinogens (Guengerich and Shimada, 1998). The SNP rs4646903 and rs1048943 of the *CYP1A1* gene, located at 15q24.1, are two importantly functional nonsynonymous SNP. In this umbrella review, relationships between rs4646903 and lung cancer risk had the strong cumulative evidence in the homozygote comparison model and the allele model. The TT genotype and T allele were associated with significantly higher risks of LC than the CC genotype and C allele, respectively. For rs1048943, associations between rs1048943 and LC risk with the strong cumulative evidence were in the recessive model. The Val/Val genotype had an increased risk of LC, compared with the Ile/Ile + Ile/Val genotype.

The *MGMT* (O-6-methylguanine-DNA methyltransferase) gene encodes a DNA repair protein that is vital to the repair of DNA damage induced by alkylating agents. Studies have demonstrated that *MGMT* plays an important role in the pathogenesis of cancers and might be a good biomarker candidate for early cancer detection (Gerson, 2004; Kaina et al., 2007). The SNP rs2308321, which is an important functional nonsynonymous SNP, is mapped to exon 7 of the *MGMT* gene at 10q26.3. The results of this umbrella review found relationships between rs2308321 and lung cancer risk with the strong cumulative evidence were in the heterozygote comparison model and the dominant model. As compared to the Ile/Ile genotype, the Ile/Val and Val/Val + Ile/Val genotypes were associated with a heightened risk of LC. Nevertheless, results of sensitivity analysis suggested that associations between rs2308321 and LC risk in the heterozygote comparison model and the dominant model were not robust or stable. Thus, the meta-analysis of the associations between rs2308321 and LC risk should be updated in the future.

The *NBS1* (Nijmegen breakage syndrome 1) gene, also known as *NBN*, *ATV*, *NBS*, *P95*, *AT-V1*, and *AT-V2*, has an important influence on the cellular response to DNA damage and maintaining chromosomal integrity, which might influence oncogenesis (Kang et al., 2005; Falck et al., 2012). SNP rs2735383 exists in the 3ʹ-untranslated region of the *NBS1* gene at 8q21.3. The current umbrella review demonstrated a strong association between rs2735383 and LC risk in the dominant model and recessive model. As compared to the GG genotype, the CC + GC genotype was associated with a high risk of LC, as was the CC genotype as compared to the GG + GC genotype.

The *TERT* (telomerase reverse transcriptase) gene, also known as *TP2*, *TRT*, *CMM9*, *EST2*, *TCS1*, *hTRT*, *DKCA2*, *DKCB4*, *hEST2*, and *PFBMFT1*, encodes the TERT protein, which is the catalytic subunit of telomerase and plays a vital role in the maintenance of telomere stability (Blackburn, 2001). Mutations to the *TERT* coding regions might influence telomere length and telomerase activity, which might further lead to substantially elevated cancer-related morbidity (Baird, 2010). The SNP rs2736098 of the *TERT* gene at 5p15.33 is a coding SNP. This umbrella review showed that there was a strong cumulative evidence on SNP rs2736098 and lung cancer risk in the heterozygote comparison model and the dominant model. In contrast to the GG genotype, the GA and GA + AA genotypes were associated with an increased risk of LC.

The *XPA* (xeroderma pigmentosum group A) gene, also known as *XP1* and *XPAC*, encodes the XPA protein, which is a DNA damage recognition and repair factor. As a zinc finger DNA binding protein, XPA is essential to nucleotide excision repair. So, a mutation to the *XPA* gene might be involved in oncogenesis (Fadda, 2016; Sugitani et al., 2016). SNP rs1800975 is localized to the 5′-untranslated region of *XPA* at 9q22.33. The current umbrella review found that rs1800975 was strongly associated with risk of LC in the recessive model. As compared to the GG + GA genotype, the AA genotype was associated with a high risk of LC.

Although the other three genes failed to be found in the LC pathway according to the prediction of the GSCALite website, they might influence the development of LC in other ways. The *ATM* (ataxia telangiectasia mutated) gene, also known as *AT1*, *ATA*, *ATC*, *ATD*, *ATE*, *ATDC*, *TEL1*, and *TELO1*, is a cancer-susceptibility gene that encodes the ATM protein, which takes part in the identification and repair of DNA damage and cell cycle regulation. Thus, a mutation to the *ATM* gene might induce not only multiple system dysfunction, but also a concomitant increase in susceptibility to LC (Kruhlak et al., 2007; Xu et al., 2017). The SNP rs664143 of the *ATM* gene is located at 11q22.3. A study of Kim et al. showed that rs664143 exists in protein-binding motifs, which may become binding sites of intronic splicing repressors or enhancers (Kim et al., 2006). The results of this umbrella review found that SNP rs664143 was strongly associated with risk of LC in the heterozygote comparison model. As compared to the GG genotype, the GA genotype was associated with a significantly increased risk of LC. However, the sample size for analyzing associations between rs664143 and LC risk was relatively small, thus further investigations are necessary.

The *CYP2E1* (cytochrome P450 family 2 subfamily E member 1) gene, also known as *CPE1*, *CYP2E*, *P450-J*, and *P450C2E*, encodes the CYP2E1 protein, which is an ethanol-inducible enzyme. CYP2E1 can metabolically activate various carcinogens, including benzene and N-nitrosamines in tobacco, and thus might play a vital role in the development of LC (Peter Guengerich and Avadhani, 2018; Guengerich, 2020). SNP rs2031920 of the *CYP2E1* gene is mapped to 10q26.3. In this umbrella review, rs2031920 was strongly associated with susceptibility to LC in the heterozygote comparison model and the dominant model. In contrast to the CC genotype, the CT and TT + CT genotypes were associated with a decreased risk of LC.

The *XRCC1* (X-ray repair cross complementing 1) gene, also known as *RCC* and *SCAR26*, encodes a DNA repair protein that can interact with DNA components at damage sites to fix DNA base damage and single-strand breaks (Hanssen-Bauer et al., 2012). Therefore, *XRCC1* plays a crucial role in protecting against tumorigenesis. SNP rs3213245 of the *XRCC1* gene is located at 19q13.31. In the present umbrella review, rs3213245 was strongly associated with susceptibility to LC in the homozygote comparison model and the recessive model. In contrast to the TT genotype, the CC genotype was associated with increased susceptibility to LC, as was the CC genotype as compared to the TT + TC genotype.

Only one SNP located on miRNA (miR-1262) was strongly associated with an increased risk of LC. A previous study reported that miR-1262 on 1p31.3 may suppress the proliferation of LC cells (Xie et al., 2017). SNP rs12740674 is located 61,743 bp downstream from miR-1262, which might map to a strong enhancer (Xie et al., 2017). The results of this umbrella review found strong associations between rs12740674 and risk of LC in the homozygote comparison model, the dominant model, and the recessive model. As compared to the CC genotype, the TT and CT + TT genotypes were associated with an increased risk of LC. Lastly, as compared to the CC + CT genotype, the TT genotype was associated with a high risk of LC.

In addition, 81 of the SNP identified in this umbrella review were not significantly correlated to LC risk in any of the five genetic models. Of these 81 SNP, 14 SNP on 12 genes had a sample size of more than 10,000, which included *APEX1* (rs1130409), *COX-2* (rs5275), *EPHX1* (rs1051740, rs2234922), *ERCC1* (rs11615), *ERCC5* (rs17655), *FASL* (rs763110), *MTHFR* (rs1801131), *NQO1* (rs1800566), *TP53* (rs1042522, rs17878362), *XPC* (rs2228001), *XRCC1* (rs25489), and *XRCC3* (rs861539). According to the calculation results obtained with Quanto 1.2.4 software (https://preventivemedicine.usc.edu/download-quanto/), 10,000 subjects provided approximately 80% statistical power if the incidence of LC was 200 per 100,000, OR was 1.15, the genetic model was the dominant model, and the MAF was 0.1. Therefore, further investigations of these 14 SNP might not be very productive.

There were certain advantages to this umbrella review. This is the first umbrella review that extracted data, rather than the results, of systematic reviews and meta-analyses to calculate and evaluate the associations between SNP and LC risk. Moreover, the combined use of the Venice criteria and FPRP increased the reliability of the assessment results. However, there were some limitations. First, subgroup analysis stratified by ethnicity was not conducted because if an umbrella review of a specific ethnicity was included, the most recent meta-analysis referring to this ethnicity must be screened again. Hence, another umbrella review of a specific ethnicity is planned in the future. Second, the quality of included meta-analyses varied to a certain extent, which might lead to data credibility issues. Third, grey literature was not included in this umbrella review.

In conclusion, this umbrella review found strong cumulative evidence of associations of 13 SNP (of 11 genes and 1 miRNA) with LC risk, which provides important references for future studies on the relationships between SNP and LC risk.

## Data Availability

The raw data supporting the conclusion of this article will be made available by the authors, without undue reservation.
