# Supplementary material for: Umbrella Review on Associations Between Single Nucleotide Polymorphisms and Lung Cancer Risk
Source: Front Mol Biosci. 2021 Sep 3;8:687105. doi: 10.3389/fmolb.2021.687105 (PMC8446528; doi:10.3389/fmolb.2021.687105)
Supplement: Supplementary file 1 [file DataSheet1.zip › Data Sheet ---Supplementary Additional file/Supplementary Additional file/Supplementary Additional file S1.docx]

**Supplementary Additional file S1. Search strategy**

1. **Search strategy for PubMed**
2. “Polymorphism, Single Nucleotide”[Mesh]
3. Nucleotide Polymorphism, Single
4. Nucleotide Polymorphisms, Single
5. Polymorphisms, Single Nucleotide
6. Single Nucleotide Polymorphisms
7. SNPs
8. Single Nucleotide Polymorphism
9. #1 OR #2 OR #3 OR #4 OR #5 OR #6 OR #7
10. “Polymorphism, Genetic”[Mesh]
11. Polymorphisms, Genetic
12. Genetic Polymorphisms
13. Genetic Polymorphism
14. Polymorphism (Genetics)
15. Polymorphisms (Genetics)
16. #9 OR #10 OR #11 OR #12 OR #13 OR #14
17. #8 OR #15
18. “Lung Neoplasms”[Mesh]
19. Pulmonary Neoplasms
20. Neoplasms, Lung
21. Lung Neoplasm
22. Neoplasm, Lung
23. Neoplasms, Pulmonary
24. Neoplasm, Pulmonary
25. Pulmonary Neoplasm
26. Lung Cancer
27. Cancer, Lung
28. Cancers, Lung
29. Lung Cancers
30. Pulmonary Cancer
31. Cancer, Pulmonary
32. Cancers, Pulmonary
33. Pulmonary Cancers
34. Cancer of the Lung
35. Cancer of Lung
36. #17 OR #18 OR #19 OR #21 OR #22 OR #23 OR #24 OR #25 OR #26 OR #27 OR #28 OR #29 OR #30 OR #31 OR #32 OR #33 OR #34
37. Systematic Review
38. Meta-Analysis
39. #36 OR #37
40. #16 AND #35 AND #38
41. **Search strategy for Web of Science**
42. TS=(Polymorphism, Single Nucleotide)
43. TS=(Nucleotide Polymorphism, Single)
44. TS=(Nucleotide Polymorphisms, Single)
45. TS=(Polymorphisms, Single Nucleotide)
46. TS=(Single Nucleotide Polymorphisms)
47. TS=(SNPs)
48. TS=(Single Nucleotide Polymorphism)
49. #1 OR #2 OR #3 OR #4 OR #5 OR #6 OR #7
50. TS=(Polymorphism, Genetic)
51. TS=(Polymorphisms, Genetic)
52. TS=(Genetic Polymorphisms)
53. TS=(Genetic Polymorphism)
54. TS=(Polymorphism (Genetics))
55. TS=(Polymorphisms (Genetics))
56. #9 OR #10 OR #11 OR #12 OR #13 OR #14
57. #8 OR #15
58. TS=(Lung Neoplasms)
59. TS=(Pulmonary Neoplasms)
60. TS=(Neoplasms, Lung)
61. TS=(Lung Neoplasm)
62. TS=(Neoplasm, Lung)
63. TS=(Neoplasms, Pulmonary)
64. TS=(Neoplasm, Pulmonary)
65. TS=(Pulmonary Neoplasm)
66. TS=(Lung Cancer)
67. TS=(Cancer, Lung)
68. TS=(Cancers, Lung)
69. TS=(Lung Cancers)
70. TS=(Pulmonary Cancer)
71. TS=(Cancer, Pulmonary)
72. TS=(Cancers, Pulmonary)
73. TS=(Pulmonary Cancers)
74. TS=(Cancer of the Lung)
75. TS=(Cancer of Lung)
76. #17 OR #18 OR #19 OR #21 OR #22 OR #23 OR #24 OR #25 OR #26 OR #27 OR #28 OR #29 OR #30 OR #31 OR #32 OR #33 OR #34
77. TS=(Systematic Review)
78. TS=(Meta-Analysis)
79. #36 OR #37
80. #16 AND #35 AND #38
81. **Search strategy for Embase**
82. ‘single nucleotide polymorphism’/exp
83. ‘polymorphism, single nucleotide’
84. ‘nucleotide polymorphism, single’
85. ‘nucleotide polymorphisms, single’
86. ‘polymorphisms, single nucleotide’
87. ‘single nucleotide polymorphisms’
88. ‘snps’
89. #1 OR #2 OR #3 OR #4 OR #5 OR #6 OR #7
90. ‘genetic polymorphism’/exp
91. ‘polymorphism, genetic’
92. ‘polymorphisms, genetic’
93. ‘genetic polymorphisms’
94. ‘polymorphism (genetics)’
95. ‘polymorphisms (genetics)’
96. #9 OR #10 OR #11 OR #12 OR #13 OR #14
97. #8 OR #15
98. ‘lung cancer’/exp
99. ‘lung Neoplasms’
100. ‘pulmonary neoplasms’
101. ‘neoplasms, lung’
102. ‘lung neoplasm’
103. ‘neoplasm, lung’
104. ‘neoplasms, pulmonary’
105. ‘neoplasm, pulmonary’
106. ‘pulmonary neoplasm’
107. ‘cancer, lung’
108. ‘cancers, lung’
109. ‘lung cancers’
110. ‘pulmonary cancer’
111. ‘cancer, pulmonary’
112. ‘cancers, pulmonary’
113. ‘pulmonary cancers’
114. ‘cancer of the lung’
115. ‘cancer of lung’
116. #17 OR #18 OR #19 OR #21 OR #22 OR #23 OR #24 OR #25 OR #26 OR #27 OR #28 OR #29 OR #30 OR #31 OR #32 OR #33 OR #34
117. ‘systematic review’
118. ‘meta-analysis’
119. #36 OR #37
120. #16 AND #35 AND #38
