# Supplementary material for: Umbrella Review on Associations Between Single Nucleotide Polymorphisms and Lung Cancer Risk
Source: Front Mol Biosci. 2021 Sep 3;8:687105. doi: 10.3389/fmolb.2021.687105 (PMC8446528; doi:10.3389/fmolb.2021.687105)
Supplement: Supplementary file 1 [file DataSheet1.zip › Data Sheet ---Supplementary Additional file/Supplementary Additional file/Supplementary Additional file S2.docx]

Supplementary Additional file S2. Basic characteristics and quality assessment of all included articles

| The first author | Year of publication | SNPs | Region | Total score |
| --- | --- | --- | --- | --- |
| Reza et al.(1) | 2020 | rs2279744 | America, Canada , China, Germany, Japan, Korea, Norway, Singapore | 4 |
| Liu et al.(2) | 2013 | rs77907221 | Belgium, China, Korea , Spain | 5 |
| Xu et al.(3) | 2017 | rs664677, rs609429, rs664982 | China, Italy, Korea | 6 |
| Yuan et al.(4) | 2016 | rs1553232011 | America, Britain, Canada, France, Germany, Iceland | 3 |
| Xie et al. 5) | 2017 | rs12740674 | China | 1 |
| Cao et al.(6) | 2014 | rs3813867 | China | 4 |
| Liu et al.(7) | 2018 | rs1695 | America, Australia, Brazil, Britain, China, Denmark, Finland, France, Germany, Greece, India, Italy, Japan, Korea, Norway, Poland, Portugal, Slovakia, Spain, Turkey | 4 |
| Han et al.(8) | 2015 | rs1051730 | America, Britain, Canada, China, Europe, Iceland, Japan, Netherlands, Norway, Spain | 4 |
| Wang et al.(9) | 2015 | rs4646994 | China, Croatia, Korea, Turkey | 6 |
| Zhu et al.(10) | 2017 | rs6983267 | China, Poland | 7 |
| Yan et al.(11) | 2017 | rs664143 | America, China, Korea | 5 |
| Wang et al.(12) | 2015 | rs20417 | China, Norway, Turkey | 4 |
| Zeng et al.(13) | 2017 | rs1065852 | China | 5 |
| Xu et al.(14) | 2020 | rs11549467 | China, Japan, Turkey | 6 |
| Liu et al.(15) | 2020 | rs12826786 | China, Turkey | 6 |
| Crosbie et al. (16) | 2008 | rs2308327, rs12268840 | Britain | 1 |
| Wang et al. (17) | 2020 | rs2910164 | China, Egypt, India, Italy, Japan, Korea, Saudi Arabia | 4 |
| Wang et al.(18) | 2018 | rs4880 | America, China, Egypt , Norway | 7 |
| Yang et al.(19) | 2017 | rs2333227 | America, Australia, China, Denmark, Finland, France, Germany, India, Japan, Korea, Norway | 7 |
| Hashemi et al.(20) | 2019 | rs2227981, rs2890658 | China, Iran | 4 |
| Chen et al.(21) | 2020 | rs4444903 | Brazil, India, Korea, Portugal | 6 |
| Li et al.(22) | 2019 | rs7975232, rs731236 | China, Poland, Tunisia, Turkey | 7 |
| Li et al.(23) | 2017 | rs8073069, rs9904341 | China, India, Korea, Turkey | 4 |
| Zhou et al.(24) | 2018 | rs2736098 | China, Korea, Poland | 5 |
| Liu et al.(25) | 2018 | rs2853669 | China, Korea | 7 |
| Chen et al.(26) | 2019 | rs1800470, rs1800469 | America, China, Korea , Portugal, Turkey | 6 |
| Peng et al.(27) | 2015 | rs4680 | America, China, Europe, Norway, Singapore | 5 |
| Chen et al.(28) | 2016 | rs1056836 | America, China, Denmark, Germany, India, Japan, Korea, Norway | 5 |
| Ye et al.(29) | 2015 | rs2031920, rs6413432 | America, China, Finland, Japan, Korea, Norway, Sweden | 4 |
| He et al.(30) | 2014 | rs776746 | Bangladesh, China, Germany | 4 |
| Hu et al.(31) | 2018 | rs2043556 | China | 5 |
| Cao et al.(32) | 2015 | rs2853676 | China, Korea, Multicenter | 6 |
| Ye et al.(33) | 2014 | rs17878362, rs1625895 | America, Europe, Slovakia, Sweden | 7 |
| He et al.(34) | 2014 | rs1800067 | America, Norway, Multicenter | 6 |
| Xie et al.(35) | 2014 | rs1800629 | America, China, Croatia, Germany , Korea, Serbia, Tunisia, Turkey | 6 |
| Qu et al.(36) | 2016 | rs578776, rs938682 | America, Britain, China, Europe, Italy, Norway | 6 |
| Yang et al.(37) | 2018 | rs3025039, rs833061 | America, Belarus , China, India, Japan, Korea, Portugal | 6 |
| Hashemi et al.(38) | 2020 | rs3834129 | China, Korea, Norway | 6 |
| Pan et al.(39) | 2020 | rs1205, rs2808630 | America, China, Japan, Netherlands | 6 |
| Chen et al.(40) | 2016 | rs1051740 | America, Australia, Britain, China, Egypt, Finland, France, Germany, India, Italy, Japan , Mexico, Spain, Turkey | 9 |
| Xu et al.(41) | 2013 | rs3212986 | America, China, France | 6 |
| Xu et al.(42) | 2019 | rs1800625 | China, Japan | 5 |
| Zhu et al.(43) | 2014 | rs3212948, rs2298881 | America, China, Europe | 5 |
| Wang et al.(44) | 2015 | rs231775 | China, Iran, Poland | 8 |
| Yu et al.(45) | 2016 | rs2234767, rs763110 | America, China, Korea, Turkey, Multicenter | 5 |
| Chen et al.(46) | 2020 | rs1801394, rs1805087, rs1801131, rs1801133 | America, China, France, Italy, Japan, Jordan, Korea, Netherlands, Poland, Serbia, Spain, Turkey | 6 |
| Zhang et al.(47) | 2016 | rs1047840 | China, Norway | 7 |
| Liu et al.(48) | 2020 | rs1799782, rs25487, rs25489, rs3213245, rs3547, rs915927, rs1136410, rs1130409, rs1760944, rs2307486, rs156641, rs20579, rs20581, rs3730931, rs439132, rs1052133, rs3219489 | America, Belgium, Britain, China, Czech Republic, Denmark, Finland, France, Germany, Greece, Hungary, India, Israel, Italy, Japan, Korea, Netherlands, Norway, Poland, Romania, Russia, Slovakia, Spain, Sweden, Turkey | 7 |
| Wu et al.(49) | 2019 | rs3136038 | China, India | 4 |
| Zhao et al.(50) | 2019 | rs189037 | China, Korea | 6 |
| Gao et al.(51) | 2020 | rs2070874, rs2243250, rs1800795, rs1800796, rs4073, rs1800872, rs1800871, rs1800896 | America, China, Denmark, France, Germany, India, Japan, Norway, Portugal, Singapore, Spain, Tunisia, Turkey | 5 |
| Zhou et al.(52) | 2020 | rs16969968 | America, Bangladesh, Britain, Canada, China, Czech Republic, France, Germany, Greece, Hungary, India , Italy , Japan, Netherlands, New Zealand, Norway, Poland, Romania, Russia, Slovakia, Spain, Sweden, Multicenter | 7 |
| Tang et al.(53) | 2017 | rs402710, rs401681 | America, Britain, China, France, Iceland, Japan , Korea, Norway, Poland | 5 |
| Tang et al.(54) | 2015 | rs31489 | America, Belgium, Britain, China, Japan, Korea, Portugal | 4 |
| Ding et al.(55) | 2019 | rs1138272 | America, Australia, Denmark, Finland, Norway, Russia | 6 |
| Dai et al.(56) | 2019 | rs2228000 | America, China, Korea | 6 |
| Li et al.(57) | 2018 | rs5275 | Brazil, China, Denmark, Europe, Kashmir, Korea, Norway | 8 |
| Wang et al.(58) | 2019 | rs689466 | Brazil, China, Denmark, Turkey | 7 |
| Li et al.(59) | 2013 | rs3569413 | India, Tunisia | 4 |
| Xu et al.(60) | 2013 | rs1048943 | America, Australia, Bangladesh, China, Britain, Germany, India, Japan, Korea, Mexico, Portugal, Singapore, Sweden, Turkey, Multicenter | 5 |
| Ji et al.(61) | 2012 | rs4646903 | America, Australia, China, Finland, Greece, India, Japan, Korea, Mexico, Norway, Portugal, Russia, Singapore, Sweden, Multicenter | 5 |
| Zheng et al.(62) | 2018 | rs2740574 | Bangladesh, Germany, Norway | 4 |
| Li et al.(63) | 2015 | rs1800440, rs1056827 | America, Germany, India, Italy, Japan, Norway | 7 |
| Mei et al.(64) | 2011 | rs238406 | America, China | 5 |
| Zhao et al.(65) | 2014 | rs3117582, rs1052486 | America, Britain, China, New Zealand, Multicenter | 5 |
| Liu et al.(66) | 2020 | rs11614913, rs2292832, rs3746444, rs4919510, rs895819 | China, Italy, Korea | 8 |
| Elliott et al. (67) | 2010 | rs4430796, rs7501939 | America, France, Iceland | 3 |
| Bu et al.(68) | 2014 | rs2470890 | Norway, America, Europe | 6 |
| Liu et al.(69) | 2017 | rs17781739, rs12587742, rs2240980 | America, Britain, Canada, France, Germany, Iceland | 5 |
| Gao et al.(70) | 2013 | rs2735383 | China | 6 |
| Geng et al.(71) | 2015 | rs20575 | America, Russia, Turkey | 5 |
| Yao et al.(72) | 2017 | rs2279115 | China | 7 |
| Li et al.(73) | 2019 | rs1001581, rs11615, rs1799793, rs13181, rs1799787, rs1800975, rs2228001, rs861539 | Unavailable | 5 |
| Hong et al.(74) | 2013 | rs1050450 | America, Denmark, Finland, Korea | 5 |
| Li et al.(75) | 2019 | rs11549465 | China, Japan, Turkey | 7 |
| Vukovic et al.(76) | 2016 | rs762551, rs2069526 | America, Belarus, Denmark, Europe, India, Japan, Norway, Spain, Tunisia | 6 |
| Li et al.(77) | 2020 | rs1799750, rs243865, rs2285053, rs35068180, rs11568818, rs3918242, rs2276109, rs2252070 | America, China, France, Lebanon, Norway, Spain, Turkey | 7 |
| Ren et al.(78) | 2016 | rs2069514 | India, Italy, Japan, Norway, Spain, Tunisia | 6 |
| Zhang et al.(79) | 2015 | rs8034191 | America, Britain, China, Norway, Poland, Multicenter | 5 |
| Lin et al.(80) | 2011 | rs1801270 | America, China, Germany, Korea, Sweden | 4 |
| Tian et al.(81) | 2014 | rs1799930, rs1799931 | China, Italy, Russia | 4 |
| He et al.(82) | 2014 | rs1805794 | China, Norway, Sweden | 5 |
| Fu et al.(83) | 2017 | rs28362491 | China, Turkey | 8 |
| Feng et al.(84) | 2018 | rs3769201, rs722864 | America, Britain, Canada, France, Germany, Iceland | 2 |
| Huang et al.(85) | 2017 | rs1800566 | America, Britain, China, Denmark, Finland, France, Germany, India, Japan, Korea, Sweden, Turkey | 8 |
| Zhou et al.(86) | 2013 | rs1042522 | America, Brazil, China, Germany, Greece, India, Iran, Italy, Japan, Korea, Poland, Singapore, Slovakia, Spain, Sweden, Turkey | 4 |
| Yin et al.(87) | 2017 | rs4646904 | America, Britain, Canada, France, Germany | 2 |
| Wang et al.(88) | 2015 | rs1801282 | China, Denmark, Norway, Singapore | 5 |
| Kang et al.(89) | 2016 | rs151606, rs12212247 | America, Britain, Canada, France, Germany, Iceland | 2 |
| Li et al.(90) | 2017 | rs1800734 | China, Korea, Slovakia | 8 |
| Yang et al.(91) | 2014 | rs2234922 | America, Austria, Finland, France, Germany, India, Italy, Norway, Spain | 9 |
| Wu et al.(92) | 2018 | rs2070600 | China | 4 |
| Chen et al.(93) | 2020 | rs1047972 | America, Turkey | 7 |
| Zhao et al.(94) | 2020 | rs699947 | Belarus, China, India, Portugal | 4 |
| Wang et al.(95) | 2020 | rs17079281 | China | 3 |
| Xu et al.(96) | 2014 | rs2273535 | America, Turkey, Multicenter | 7 |
| Xiao et al.(97) | 2014 | rs9282861 | America, China, India, Japan , Turkey | 6 |
| Zhou et al.(98) | 2017 | rs2453176 | America, Britain, Canada, France, Germany, Iceland | 4 |
| Xu et al.(99) | 2013 | rs1143634 | America, China, Japan, Norway | 8 |
| Yang et al.(100) | 2015 | rs2735940 | America, China, Iceland, Korea | 6 |
| He et al.(101) | 2019 | rs10069690 | China, Multicenter | 6 |
| Luo et al.(102) | 2013 | rs2066853 | China, France, Japan, Korea | 7 |
| Pan et al.(103) | 2019 | rs662 | China, Korea, Turkey | 8 |
| Xie et al.(104) | 2020 | rs2853677 | China | 7 |
| Xiao et al.(105) | 2014 | rs6495309 | China | 7 |
| Dai et al.(106) | 2019 | rs2240308 | China, India, Japan, Turkey | 9 |
| Duan et al.(107) | 2020 | rs1544410 | China, Poland, Tunisia, Turkey | 9 |
| Lian et al.(108) | 2019 | rs4245739 | China, Norway | 7 |
| Zhou et al.(109) | 2013 | rs9344 | America, Britain, China, Europe, India | 6 |
| Chen et al.(110) | 2017 | rs2424913, rs1569686 | China | 5 |
| Du et al.(111) | 2013 | rs2308321, rs1803865 | America, Britain, China, Korea | 7 |
| Sheng et al.(112) | 2018 | rs12917 | America, China, Korea, Norway | 6 |
| Liu et al.(113) | 2016 | rs1800624 | China | 5 |
| Du et al.(114) | 2018 | rs1447295 | America, China | 7 |
| Wang et al.(115) | 2015 | rs931794 | America, China, France, Japan | 7 |
| Li et al.(116) | 2017 | rs2736100 | America, Britain, China, France, Japan, Korea, Poland | 4 |
| Liu et al.(117) | 2014 | rs2352028 | America, Britain, China, Germany, Iceland | 5 |
| Ban et al.(118) | 2015 | rs2010963 | Canada, China, Korea | 8 |
| Huang et al.(119) | 2017 | rs1047768 | America, China, Norway | 5 |
| Zhao et al.(120) | 2018 | rs17655 | America, China, Japan, Multicenter | 6 |

References

1. Reza HA, Anamika WJ, Chowdhury MMK, Mostafa MG, Uddin MA. A cohort study on the association of MDM2 SNP309 with lung cancer risk in Bangladeshi population. The Korean journal of internal medicine. 2020;35(3):672-81.

2. Liu C, Yin Q, Hu J, Li L, Zhang Y, Wang Y. A meta-analysis of evidences on XPC polymorphisms and lung cancer susceptibility. Tumour biology : the journal of the International Society for Oncodevelopmental Biology and Medicine. 2013;34(2):1205-13.

3. Xu Y, Gao P, Lv X, Zhang L, Li W, Zhang J. A meta-analysis of the relationship between ataxia-telangiectasia mutated gene polymorphisms and lung cancer susceptibility. Pathology, research and practice. 2017;213(9):1152-9.

4. Yuan H, Liu H, Liu Z, Owzar K, Han Y, Su L, et al. A Novel Genetic Variant in Long Non-coding RNA Gene NEXN-AS1 is Associated with Risk of Lung Cancer. Scientific reports. 2016;6.

5. Xie K, Chen M, Zhu M, Wang C, Qin N, Liang C, et al. A polymorphism in miR-1262 regulatory region confers the risk of lung cancer in Chinese population. International journal of cancer. 2017;141(5):958-66.

6. Cao L, Lin J, He B, Wang H, Rao J, Liu Y, et al. A regulatory variant in CYP2E1 affects the risk of lung squamous cell carcinoma. Tumor Biology. 2014;35(1):455-62.

7. Liu WZ, Sun Y, Feng X, Bi XH, Liu T, Zhou HF. An updated meta-analysis for association of glutathione S-transferase P1 gene polymorphism with the susceptibility of lung cancer. Journal of cancer research and therapeutics. 2018;14(12):S1084-S90.

8. Han Z, Jiang Q, Zhang T, Wu X, Ma R, Wang J, et al. Analyzing large-scale samples confirms the association between the rs1051730 polymorphism and lung cancer susceptibility. Scientific reports. 2015;5.

9. Wang N, Yang D, Ji B, Li J. Angiotensin-converting enzyme insertion/deletion gene polymorphism and lung cancer risk: A meta-analysis. Journal of the Renin-Angiotensin-Aldosterone System. 2015;16(1):189-94.

10. Zhu M, Wen X, Liu X, Wang Y, Liang C, Tu J. Association between 8q24 rs6983267 polymorphism and cancer susceptibility: A meta-analysis involving 170,737 subjects. Oncotarget. 2017;8(34):57421-39.

11. Yan Z, Tong X, Ma Y, Liu S, Yang L, Yang X, et al. Association between ATM gene polymorphisms, lung cancer susceptibility and radiation-induced pneumonitis: A meta-analysis. BMC pulmonary medicine. 2017;17(1).

12. Wang W, Fan X, Zhang Y, Yang Y, Yang S, Li G. Association between COX-2 polymorphisms and lung cancer risk. Medical Science Monitor. 2015;21:3740-7.

13. Zeng J, Li J, Bao M, Long Y, Li G, Luo Y. Association between CYP2D6 polymorphisms and lung cancer risk: An up-date meta-analysis. International Journal of Clinical and Experimental Medicine. 2017;10(3):4508-17.

14. Xu S, Ying K. Association between HIF-1 alpha gene polymorphisms and lung cancer A meta-analysis. Medicine. 2020;99(24).

15. Liu X, Zhao Y, Li Y, Lin F, Zhang J. Association between HOTAIR genetic polymorphisms and cancer susceptibility: A meta-analysis involving 122,832 subjects. Genomics. 2020;112(5):3036-55.

16. Crosbie PAJ, McGown G, Thorncroft MR, O'Donnell PNS, Barber PV, Lewis SJ, et al. Association between lung cancer risk and single nucleotide polymorphisms in the first intron and codon 178 of the DNA repair gene, O-6-alkylguanine-DNA alkyltransferase. International journal of cancer. 2008;122(4):791-5.

17. Wang D, Wang Y, Lin Z, Cai L. Association between miRNA-146a polymorphism and lung cancer susceptibility: A meta-analysis involving 6506 cases and 6576 controls. Gene. 2020;757.

18. Wang P, Zhu Y, Xi S, Li S, Zhang Y. Association between MnSOD Val16Ala Polymorphism and Cancer Risk: Evidence from 33,098 Cases and 37,831 Controls. Disease markers. 2018.

19. Yang WJ, Wang MY, Pan FZ, Shi C, Cen H. Association between MPO-463G > A polymorphism and cancer risk: evidence from 60 case-control studies. World journal of surgical oncology. 2017;15(1):144.

20. Hashemi M, Karami S, Sarabandi S, Moazeni-Roodi A, Malecki A, Ghavami S, et al. Association between PD-1 and PD-L1 Polymorphisms and the Risk of Cancer: A Meta-Analysis of Case-Control Studies. Cancers. 2019;11(8).

21. Chen Q, Zheng Y, Wu B, Chen X, Ge P, Wang P. Association between polymorphisms of epidermal growth factor 61 and susceptibility of lung cancer A meta-analysis. Medicine. 2020;99(17).

22. Li M, Liu X, Liu N, Yang T, Shi P, He R, et al. Association between Polymorphisms of Vitamin D Receptor and Lung Cancer Susceptibility: Evidence from an Updated Meta-analysis. Journal of Cancer. 2019;10(16):3639-49.

23. Li H, Ma B, Wang D, Li M, Yuan S. Association between survivin gene -31G/C and 9194A/G polymorphisms and lung cancer susceptibility: Meta-analysis of six studies. International Journal of Clinical and Experimental Medicine. 2017;10(3):4360-7.

24. Zhou M, Jiang B, Xiong M, Zhu X. Association between TERT rs2736098 polymorphisms and cancer risk-A meta-analysis. Frontiers in Physiology. 2018;9(APR).

25. Liu Z, Wang T, Wu Z, Zhang K, Li W, Yang J, et al. Association between TERT rs2853669 polymorphism and cancer risk: A meta-analysis of 9,157 cases and 11,073 controls. PloS one. 2018;13(3).

26. Chen G, Hu C, Lai P, Song Y, Xiu M, Zhang H, et al. Association between TGF-beta 1 rs1982073/rs1800469 polymorphism and lung cancer susceptibility An updated meta-analysis involving 7698 cases and controls. Medicine. 2019;98(47).

27. Peng S, Tong X, Liu S, Feng Y, Fan H. Association between the COMT 158 G/A polymorphism and lung cancer risk: A meta-analysis. International Journal of Clinical and Experimental Medicine. 2015;8(10):17739-47.

28. Chen P-F, He X-F, Huang G-H, Wang W, Qiu Z-H. Association Between the CYP1B1 Polymorphisms and Lung Cancer Risk: A Meta-Analysis. Technology in cancer research & treatment. 2016;15(5):NP73-NP82.

29. Ye X-H, Song L, Peng L, Bu Z, Yan S-X, Feng J, et al. Association between the CYP2E1 polymorphisms and lung cancer risk: a meta-analysis. Molecular Genetics and Genomics. 2015;290(2):545-58.

30. He X-F, Liu Z-Z, Xie J-J, Wang W, Du Y-P, Chen Y, et al. Association between the CYP3A4 and CYP3A5 polymorphisms and cancer risk: a meta-analysis and meta-regression. Tumor Biology. 2014;35(10):9859-77.

31. Hu M, Yu Z, Luo D, Zhang H, Li J, Liang F, et al. Association between the polymorphism in miR-605 and cancer susceptibility: A PRISMA-compliant meta-analysis. Clinical Laboratory. 2018;64(11-12):1917-24.

32. Cao J-L, Yuan P, Abuduwufuer A, Lv W, Yang Y-H, Hu J. Association between the TERT Genetic Polymorphism rs2853676 and Cancer Risk: Meta-Analysis of 76 108 Cases and 134 215 Controls. PloS one. 2015;10(6).

33. Ye X-H, Bu Z-B, Feng J, Peng L, Liao X-B, Zhu X-L, et al. Association between the TP53 polymorphisms and lung cancer risk: a meta-analysis. Molecular biology reports. 2014;41(1):373-85.

34. He X-F, Liu L-R, Wei W, Liu Y, Su J, Wang S-L, et al. Association between the XPG Asp1104His and XPF Arg415Gln Polymorphisms and Risk of Cancer: A Meta-Analysis. PloS one. 2014;9(5).

35. Xie H, Yao H, Huo Y, Li N, Cheng Y. Association between TNF-α gene 308G>A polymorphism and lung cancer risk: a meta-analysis. Tumour biology : the journal of the International Society for Oncodevelopmental Biology and Medicine. 2014;35(10):9693-9.

36. Qu X, Wang K, Dong W, Shen H, Wang Y, Liu Q, et al. Association between two CHRNA3 variants and susceptibility of lung cancer: a meta-analysis. Scientific reports. 2016;6.

37. Yang F, Qin Z, Shao C, Liu W, Ma L, Shu Y, et al. Association between VEGF Gene Polymorphisms and the Susceptibility to Lung Cancer: An Updated Meta-Analysis. BioMed research international. 2018;2018.

38. Hashemi M, Aftabi S, Moazeni-Roodi A, Sarani H, Wiechec E, Ghavami S. Association of CASP8 polymorphisms and cancer susceptibility: A meta-analysis. European Journal of Pharmacology. 2020;881.

39. Pan HY, Mi YY, Xu K, Zhang Z, Wu H, Zhang W, et al. Association of C-reactive protein (CRP) rs1205 and rs2808630 variants and risk of cancer. Journal of Cellular Physiology. 2020.

40. Chen Y, Tian J, Qin F, Wang P. Association of EPHX1 Tyr113His polymorphism with the susceptibility to lung cancer. International Journal of Clinical and Experimental Medicine. 2016;9(9):17703-14.

41. Xu TP, Shen H, Liu LX, Shu YQ. Association of ERCC1-C118T and -C8092A polymorphisms with lung cancer risk and survival of advanced-stage non-small cell lung cancer patients receiving platinum-based chemotherapy: A pooled analysis based on 39 reports. Gene. 2013;526(2):265-74.

42. Xu Y, Lu Z, Shen N, Wang X. Association of RAGE rs1800625 Polymorphism and Cancer Risk: A Meta-Analysis of 18 Case-Control Studies. Medical Science Monitor. 2019;25:7026-34.

43. Zhu J, Hua RX, Jiang J, Zhao LQ, Sun X, Luan J, et al. Association studies of ERCC1 polymorphisms with lung cancer susceptibility: A systematic review and meta-analysis. PloS one. 2014;9(5).

44. Wang L, Jiang Z, Qiu H, Tang W, Duan T, Wang L. Associations between CTLA-4 +49 A/G (Rs231775) polymorphism and cancer risk: A meta-analysis based on 52 case-control studies. International Journal of Clinical and Experimental Medicine. 2015;8(5):6835-51.

45. Yu X, Li Y, Yu Y, Lei J, Wan G, Cao F. Associations between FAS rs2234767 and FASL rs763110 polymorphisms and the risk of lung cancer: A meta-analysis of 39,736 subjects. OncoTargets and Therapy. 2016;9:2049-56.

46. Chen F, Wen T, Lv Q, Liu F. Associations between Folate Metabolism Enzyme Polymorphisms and Lung Cancer: A Meta-Analysis. Nutrition and Cancer. 2020;72(7):1211-8.

47. Zhang M, Zhao D, Yan C, Zhang L, Liang C. Associations between Nine Polymorphisms in EXO1 and Cancer Susceptibility: A Systematic Review and Meta-Analysis of 39 Case-control Studies. Scientific reports. 2016;6.

48. Liu S, Xiao Y, Hu C, Li M. Associations between polymorphisms in genes of base excision repair pathway and lung cancer risk. Translational Cancer Research. 2020;9(4):2780-800.

49. Wu H, Li S, Hu X, Qin W, Wang Y, Sun T, et al. Associations of mRNA expression of DNA repair genes and genetic polymorphisms with cancer risk: a bioinformatics analysis and meta-analysis. Journal of Cancer. 2019;10(16):3593-607.

50. Zhao ZL, Xia L, Zhao C, Yao J. ATM rs189037 (G > A) polymorphism increased the risk of cancer: an updated meta-analysis. BMC medical genetics. 2019;20(1):28.

51. Gao J, Ying Y, Wang J, Cui Y, Zhang W. Certain interleukin polymorphisms might influence predisposition to lung cancer: A meta-analysis of 35 published studies. IUBMB Life. 2020;72(5):957-64.

52. Zhou W, Zhu W, Tong X, Ming S, Ding Y, Li Y, et al. CHRNA5 rs16969968 polymorphism is associated with lung cancer risk: A meta-analysis. Clinical Respiratory Journal. 2020;14(6):505-13.

53. Tang J, Hu C, Mei H, Peng L, Li H. CLPTM1L gene rs402710 (C > T) and rs401681 (C > T) polymorphisms associate with decreased cancer risk: A meta-analysis. Oncotarget. 2017;8(60):102446-57.

54. Tang M, Bian X, Zhao Q. CLPTM1L polymorphism and lung cancer risk. International Journal of Clinical and Experimental Medicine. 2015;8(3):3895-900.

55. Ding F, Li JP, Zhang Y, Qi GH, Song ZC, Yu YH. Comprehensive Analysis of the Association between the rs1138272 Polymorphism of the GSTP1 gene and cancer susceptibility. Frontiers in Physiology. 2019;10(JAN).

56. Dai Y, Song Z, Zhang J, Gao W. Comprehensive assessment of the association between XPC rs2228000 and cancer susceptibility based on 26835 cancer cases and 37069 controls. Bioscience reports. 2019;39(12).

57. Li J, Lu X, Zou X, Jiang Y, Yao J, Liu H, et al. COX-2 rs5275 and rs689466 polymorphism and risk of lung cancer: A PRISMA-compliant meta-analysis. Medicine (United States). 2018;97(35).

58. Wang J, Li G-M, Wang X-D, Zhao F-Y, Li Y, Sun J-G, et al. COX-2 rs689466 polymorphism correlates with increased lung cancer risk. International Journal of Clinical and Experimental Medicine. 2019;12(3):2538-48.

59. Li Z, Liu X, Sun N, Guo Z, Ren C, Li J. Current evidence on the relationship between three polymorphisms in the CYP1A2 gene and the risk of cancer. European Journal of Cancer Prevention. 2013;22(6):607-19.

60. Xu C-h, Wang Q, Qian Q, Zhan P, Yu L-K. CYP1A1 exon7 polymorphism is associated with lung cancer risk among the female population and among smokers: a meta-analysis. Tumor Biology. 2013;34(6):3901-11.

61. Ji YN, Wang Q, Lin XQ, Suo LJ. CYP1A1 MspI polymorphisms and lung cancer risk: An updated meta-analysis involving 20,209 subjects. Cytokine. 2012;59(2):324-34.

62. Zheng Y, Xu Y, Zhou B-Y, Sun L, Yu P-B, Zhang L, et al. CYP3A4*1B Polymorphism and Cancer Risk: A Meta-Analysis Based on 55 Case-control Studies. Annals of Clinical and Laboratory Science. 2018;48(4):538-45.

63. Li C, Long B, Qin X, Li W, Zhou Y. Cytochrome P1B1 (CYP1B1) polymorphisms and cancer risk: A meta-analysis of 52 studies. Toxicology. 2015;327:77-86.

64. Mei C-r, Luo M, Li H-m, Deng W-j, Zhou Q-h. DNA repair gene polymorphisms in the nucleotide excision repair pathway and lung cancer risk: A meta-analysis. Chinese Journal of Cancer Research. 2011;23(2):79-91.

65. Zhao J, Wang H, Hu W, Jin Y. Effect of HLA-B-associated transcript 3 polymorphisms on lung cancer risk: A meta-analysis. Medical Science Monitor. 2014;20:2461-5.

66. Liu G, Tian J, Zuo C, Li Y, Fu K, Chen H. Epidemiological evidence for associations between variants in microRNA or biosynthesis genes and lung cancer risk. Cancer Medicine. 2020;9(5):1937-50.

67. Elliott KS, Zeggini E, McCarthy MI, Gudmundsson J, Sulem P, Stacey SN, et al. Evaluation of Association of HNF1B Variants with Diverse Cancers: Collaborative Analysis of Data from 19 Genome-Wide Association Studies. PloS one. 2010;5(5).

68. Bu Z-B, Ye M, Cheng Y, Wu W-Z. Four Polymorphisms in the Cytochrome P450 1A2 (CYP1A2) Gene and Lung Cancer Risk: a Meta-analysis. Asian Pacific Journal of Cancer Prevention. 2014;15(14):5673-9.

69. Liu H, Liu Z, Wang Y, Stinchcombe TE, Owzar K, Han Y, et al. Functional variants in DCAF4 associated with lung cancer risk in European populations. Carcinogenesis. 2017;38(5):541-51.

70. Gao P, Ma N, Li M, Tian QB, Liu DW. Functional variants in NBS1 and cancer risk: Evidence from a meta-analysis of 60 publications with 111 individual studies. Mutagenesis. 2013;28(6):683-97.

71. Geng P, Li J, Wang N, Liao Y, Ou J, Sa R, et al. Genetic association between TRAIL-R1 Thr209Arg and cancer susceptibility. Scientific reports. 2015;5.

72. Yao Z, Yang B, Liu Z, Li W, He Q, Peng X. Genetic polymorphisms of Bcl-2 promoter in cancer susceptibility and prognosis: a meta-analysis. Oncotarget. 2017;8(15):25270-8.

73. Li W, Zhang M, Huang C, Meng J, Yin X, Sun G. Genetic variants of DNA repair pathway genes on lung cancer risk. Pathology Research and Practice. 2019;215(10).

74. Hong Z, Tian C, Zhang X. GPX1 gene Pro200Leu polymorphism, erythrocyte GPX activity, and cancer risk. Molecular biology reports. 2013;40(2):1801-12.

75. Li HN, He T, Zha YJ, Du F, Liu J, Lin HR, et al. HIF-1α rs11549465 C>T polymorphism contributes to increased cancer susceptibility: Evidence from 49 studies. Journal of Cancer. 2019;10(24):5955-63.

76. Vukovic V, Ianuale C, Leoncini E, Pastorino R, Gualano MR, Amore R, et al. Lack of association between polymorphisms in the CYP1A2 gene and risk of cancer: Evidence from meta-analyses. BMC cancer. 2016;16(1).

77. Li X, Liu C, Ran R, Liu G, Yang Y, Zhao W, et al. Matrix metalloproteinase family gene polymorphisms and lung cancer susceptibility: An updated meta-analysis. Journal of Thoracic Disease. 2020;12(3):349-62.

78. Ren J, He BZ, Zhang TS, Lu SP, Yan T. Meta-analysis of correlation between the CYP1A2 -3860 G > A polymorphism and lung cancer risk. Genetics and Molecular Research. 2016;15(2).

79. Zhang L, Jin TB, Gao Y, Wang HJ, Yang H, Feng T, et al. Meta-analysis of the association between the rs8034191 polymorphism in AGPHD1 and lung cancer risk. Asian Pacific journal of cancer prevention : APJCP. 2015;16(7):2713-7.

80. Lin G, Fang F, Yu XJ, Yu L. Meta-analysis of the relationship between p21 Ser31Arg polymorphism and lung cancer susceptibility. Genetics and Molecular Research. 2011;10(4):2449-56.

81. Tian F-S, Shen L, Ren Y-W, Zhang Y, Yin Z-H, Zhou B-S. N-Acetyltransferase 2 Gene Polymorphisms are Associated with Susceptibility to Cancer: a Meta-analysis. Asian Pacific Journal of Cancer Prevention. 2014;15(14):5621-6.

82. He Y-Z, Chi X-S, Zhang Y-C, Deng X-B, Wang J-R, Lv W-Y, et al. NBS1 Glu185Gln polymorphism and cancer risk: update on current evidence. Tumor Biology. 2014;35(1):675-87.

83. Fu W, Zhuo Z-J, Chen Y-C, Zhu J, Zhao Z, Jia W, et al. NFKB1-94insertion/deletion ATTG polymorphism and cancer risk: Evidence from 50 case-control studies. Oncotarget. 2017;8(6):9806-22.

84. Feng Y, Wang Y, Liu H, Liu Z, Mills C, Owzar K, et al. Novel genetic variants in the P38MAPK pathway gene ZAK and susceptibility to lung cancer. Molecular carcinogenesis. 2018;57(2):216-24.

85. Huang J, Lin H, Wu X, Jin W, Zhang Z. NQO1 C609T polymorphism and lung cancer susceptibility: Evidence from a comprehensive meta-analysis. Oncotarget. 2017;8(60):102301-9.

86. Zhou C, Chen H, Wang A. P53 codon 72 polymorphism and lung cancer risk: Evidence from 27,958 subjects. Tumor Biology. 2013;34(5):2961-9.

87. Yin J, Liu H, Liu Z, Owzar K, Han Y, Su L, et al. Pathway-analysis of published genome-wide association studies of lung cancer: A potential role for the CYP4F3 locus. Molecular carcinogenesis. 2017;56(6):1663-72.

88. Wang Y, Chen Y, Jiang H, Tang W, Kang M, Liu T, et al. Peroxisome proliferator-activated receptor gamma (PPARG) rs1801282 C>G polymorphism is associated with cancer susceptibility in asians: An updated meta-analysis. International Journal of Clinical and Experimental Medicine. 2015;8(8):12661-73.

89. Kang X, Liu H, Onaitis MW, Liu Z, Owzar K, Han Y, et al. Polymorphisms of the centrosomal gene (FGFR1OP) and lung cancer risk: a meta-analysis of 14 463 cases and 44 188 controls. Carcinogenesis. 2016;37(3):280-9.

90. Li S, Zheng Y, Tian T, Wang M, Liu X, Liu K, et al. Pooling-analysis on hMLH1 polymorphisms and cancer risk: Evidence based on 31,484 cancer cases and 45,494 cancer-free controls. Oncotarget. 2017;8(54):93063-78.

91. Yang X, Wang Y, Wang G. Quantitative assessment of the influence of EPHX1 gene polymorphisms and cancer risk: a meta-analysis with 94,213 subjects. Journal of Experimental & Clinical Cancer Research. 2014;33.

92. Wu S, Mao L, Li Y, Yin Y, Yuan W, Chen Y, et al. RAGE may act as a tumour suppressor to regulate lung cancer development. Gene. 2018;651:86-93.

93. Chen G, Hu C, Song Y, Xiu M, Zhang Y, Lai P, et al. Relationship between aurora-A V57I polymorphism and the risk of cancer: A meta-analysis and trial sequential analysis. Journal of Cancer. 2020;11(11):3225-34.

94. Zhao H-l, Yu J-h, Huang L-s, Li P-z, Lao M, Zhu B, et al. Relationship between vascular endothelial growth factor-2578C > a gene polymorphism and lung cancer risk: a meta-analysis. BMC medical genetics. 2020;21(1).

95. Wang Y, Ma R, Liu B, Kong J, Lin H, Yu X, et al. SNP rs17079281 decreases lung cancer risk through creating an YY1-binding site to suppress DCBLD1 expression. Oncogene. 2020;39(20):4092-102.

96. Xu L, Zhou X, Jiang F, Xu L, Yin R. STK15 rs2273535 polymorphism and cancer risk: A meta-analysis of 74,896 subjects. Cancer epidemiology. 2014;38(2):111-7.

97. Xiao J, Zheng Y, Zhou Y, Zhang P, Wang J, Shen F, et al. Sulfotransferase SULT1A1 Arg213His Polymorphism with Cancer Risk: A Meta-Analysis of 53 Case-Control Studies. PloS one. 2014;9(9).

98. Zhou F, Wang Y, Liu H, Ready N, Han Y, Hung RJ, et al. Susceptibility loci of CNOT6 in the general mRNA degradation pathway and lung cancer risk-A re-analysis of eight GWASs. Molecular carcinogenesis. 2017;56(4):1227-38.

99. Xu J, Yin Z, Cao S, Gao W, Liu L, Yin Y, et al. Systematic Review and Meta-Analysis on the Association between IL-1B Polymorphisms and Cancer Risk. PloS one. 2013;8(5).

100. Yang H, Li J, Tang R, Liu Y, Shao Y, Huang Q, et al. Telomere reverse transcriptase (TERT) rs2735940 increases cancer risk. Medical Science Monitor. 2015;21:612-6.

101. He G, Song T, Zhang Y, Chen X, Xiong W, Chen H, et al. TERT rs10069690 polymorphism and cancers risk: A meta-analysis. Molecular genetics & genomic medicine. 2019;7(10):e00903.

102. Luo C, Zou P, Ji G, Gu A, Zhao P, Zhao C. The aryl hydrocarbon receptor (AhR) 1661G>A polymorphism in human cancer: A meta-analysis. Gene. 2013;513(1):225-30.

103. Pan X, Huang L, Li M, Mo D, Liang Y, Liu Z, et al. The Association between PON1 (Q192R and L55M) Gene Polymorphisms and Risk of Cancer: A Meta-Analysis Based on 43 Studies. BioMed research international. 2019;2019.

104. Xie Y, Zhu J, Fu Y, Guo X, Huang J, Liu Z. The association between tert RS2853677 (A > G) and cancer risk: A meta-analysis. International Journal of Clinical and Experimental Medicine. 2020;13(3):1323-33.

105. Xiao M, Chen L, Wu X, Wen F. The association between the rs6495309 polymorphism in CHRNA3 gene and lung cancer risk in Chinese: a meta-analysis. Scientific reports. 2014;4.

106. Dai F, Zhu LJ, Zhang W, Mi YY, Sun HY, Zhang LF, et al. The association between three AXIN2 variants and cancer risk. Journal of cellular biochemistry. 2019;120(9):15561-71.

107. Duan GQ, Zheng X, Li WK, Zhang W, Li Z, Tan W. The Association between VDR and GC Polymorphisms and Lung Cancer Risk: A Systematic Review and Meta-Analysis. Genetic testing and molecular biomarkers. 2020;24(5):285-95.

108. Lian T, Zhu J, He J, Li C, Tang R, Jiang L, et al. The associations between mdm4 rs4245739 a>c polymorphism and cancer risk: A meta-analysis. International Journal of Clinical and Experimental Medicine. 2019;12(8):10411-21.

109. Zhou C, An H, Hu M, Liu Q, Geng P, Xu J, et al. The cyclin D1 (CCND1) G870A polymorphism and lung cancer susceptibility: A meta-analysis. Tumor Biology. 2013;34(6):3831-7.

110. Chen B, Wang J, Gu X, Zhang J, Zhang J, Feng X. The DNMT3B -579G>T Polymorphism Is Significantly Associated With the Risk of Gastric Cancer but not Lung Cancer in Chinese Population. Technology in Cancer Research and Treatment. 2017;16(6):1259-65.

111. Du L, Wang H, Xiong T, Ma Y, Yang J, Huang J, et al. The polymorphisms in the MGMT gene and the risk of cancer: a meta-analysis. Tumor Biology. 2013;34(5):3227-37.

112. Sheng Z, Kang M, Wang H. The potential role of MGMT rs12917 polymorphism in cancer risk: An updated pooling analysis with 21010 cases and 34018 controls. Bioscience reports. 2018;38(5).

113. Liu S, Tong X, He M, Fu X, Zhang Y, Fan H. The receptor for advanced glycation end products gene polymorphisms contribute to cancer susceptibility: Evidence from meta-analysis. International Journal of Clinical and Experimental Medicine. 2016;9(3):5867-79.

114. Du P, Zhu J, He C, Hu G, Li S, Ye MY, et al. The rs1447295 polymorphism in the 8q24 gene contributes to cancer risk, especially prostate cancer: A meta-analysis. International Journal of Clinical and Experimental Medicine. 2018;11(12):13115-34.

115. Wang Q, Ke J, Song Q, Hu W, Lu X, Wang Z, et al. The SNP rs931794 in 15q25.1 is associated with lung cancer risk: A hospital-based case-control study and meta-analysis. PloS one. 2015;10(6).

116. Li H, Xu Y, Mei H, Peng L, Li X, Tang J. The TERT rs2736100 polymorphism increases cancer risk: A meta-analysis. Oncotarget. 2017;8(24):38693-705.

117. Liu L, Zhong R, Zou L, Fu J, Zhu B, Chen W, et al. Variants in the 5'-upstream region of GPC5 confer risk of lung cancer in never smokers. Cancer epidemiology. 2014;38(1):66-72.

118. Ban JY, Shin JI, Oh CH. Vascular endothelial growth factor-634 G/C polymorphism and risk of cancer: An updated meta-analysis. Genetics and Molecular Research. 2015;14(4):13906-14.

119. Huang J, Liu X, Tang L-L, Long J-T, Zhu J, Hua R-X, et al. XPG gene polymorphisms and cancer susceptibility: evidence from 47 studies. Oncotarget. 2017;8(23):37263-77.

120. Zhao J, Chen S, Zhou H, Zhang T, Liu Y, He J, et al. XPG rs17655 G > C polymorphism associated with cancer risk: evidence from 60 studies. Aging-Us. 2018;10(5):1073-88.
