# Supplementary material for: Umbrella Review on Associations Between Single Nucleotide Polymorphisms and Lung Cancer Risk
Source: Front Mol Biosci. 2021 Sep 3;8:687105. doi: 10.3389/fmolb.2021.687105 (PMC8446528; doi:10.3389/fmolb.2021.687105)
Supplement: Supplementary file 1 [file DataSheet1.zip › Data Sheet ---Supplementary Additional file/Supplementary Additional file/Supplementary Additional file S3.docx]

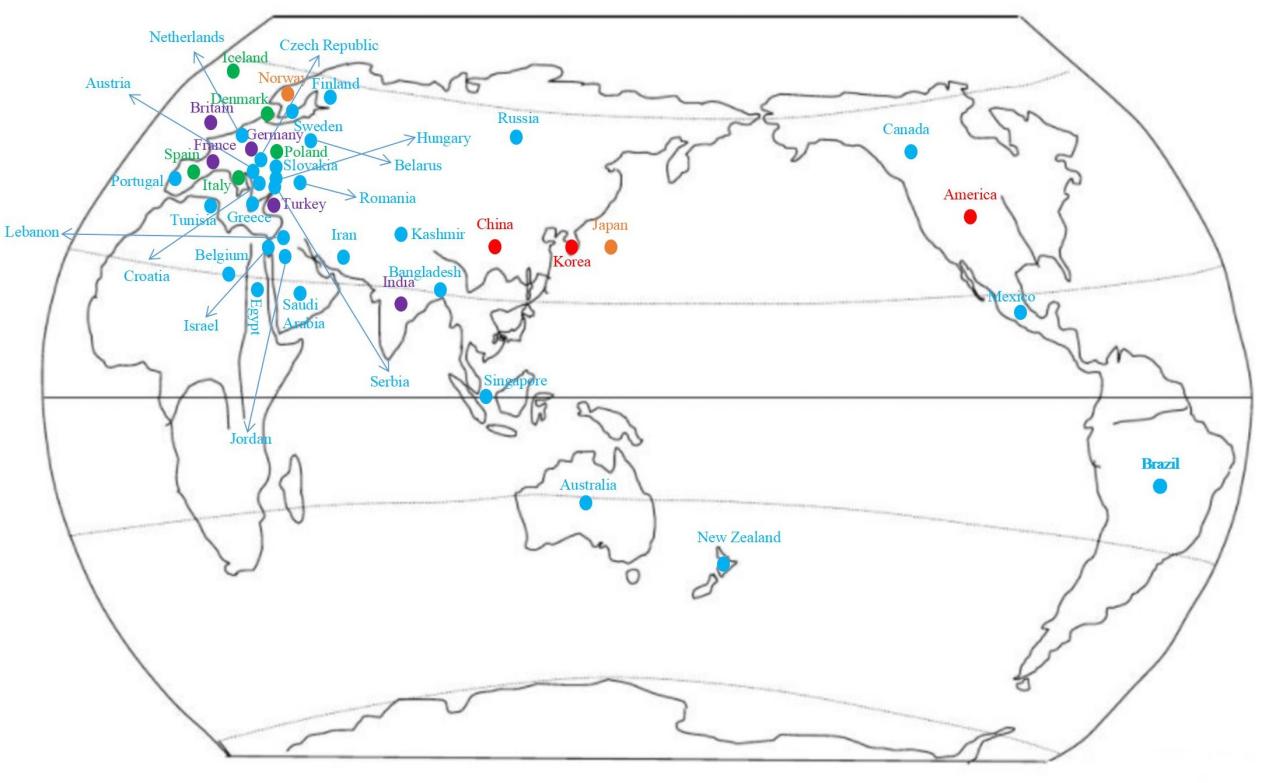
Supplementary Additional file S3. A map of the world where subjects taken for the study came from.

Different colors represent the number of times the different regions were involved in the 120 articles included in this study: red, >40; orange, 31-40; purple, 21-30; green, 11-20; blue, ≤10.

Multicenter studies are not considered in this map.
