# Supplementary material for: Umbrella Review on Associations Between Single Nucleotide Polymorphisms and Lung Cancer Risk
Source: Front Mol Biosci. 2021 Sep 3;8:687105. doi: 10.3389/fmolb.2021.687105 (PMC8446528; doi:10.3389/fmolb.2021.687105)
Supplement: Supplementary file 1 [file DataSheet1.zip › Data Sheet ---Supplementary Additional file/Supplementary Additional file/Supplementary Additional file S7.docx]

Supplementary Additional file S7. Sensitivity analyses for SNPs and lung cancer risk


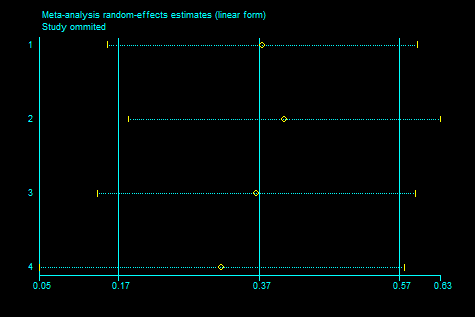


Supplementary Additional file S7. Figure1 Sensitivity analysis for rs664143 and lung cancer risk in the heterozygote comparison model


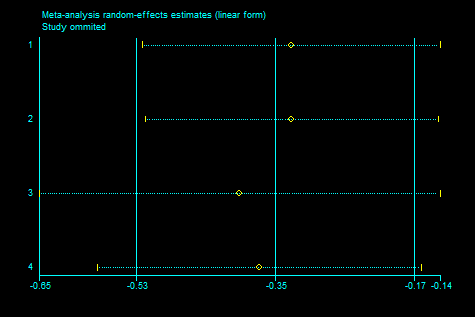


Supplementary Additional file S7. Figure2 Sensitivity analysis for rs2240308 and lung cancer risk in the dominant model


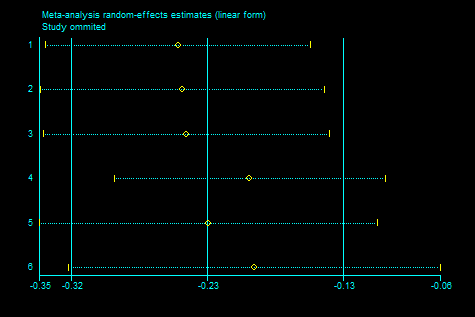


Supplementary Additional file S7. Figure3 Sensitivity analysis for rs938682 and lung cancer risk in the allele model


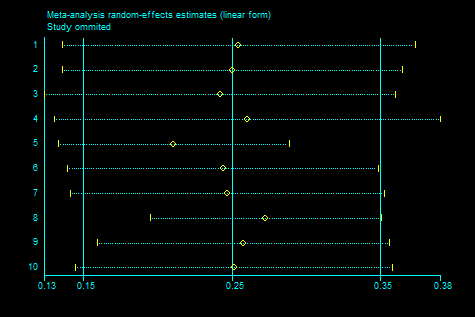


Supplementary Additional file S7. Figure4 Sensitivity analysis for rs31489 and lung cancer risk in the homozygote comparison model


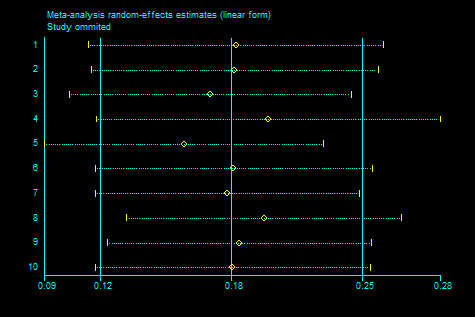


Supplementary Additional file S7. Figure5 Sensitivity analysis for rs31489 and lung cancer risk in the dominant model


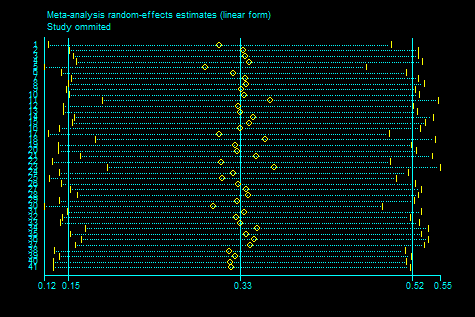


Supplementary Additional file S7. Figure6 Sensitivity analysis for rs4646903 and lung cancer risk in the homozygote comparison model


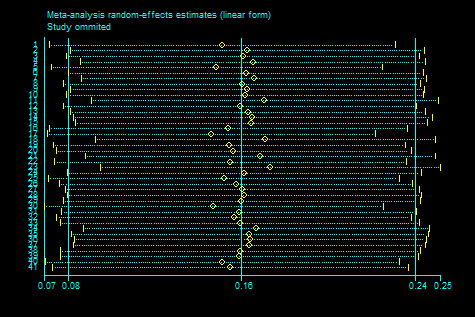


Supplementary Additional file S7. Figure7 Sensitivity analysis for rs4646903 and lung cancer risk in the allele model


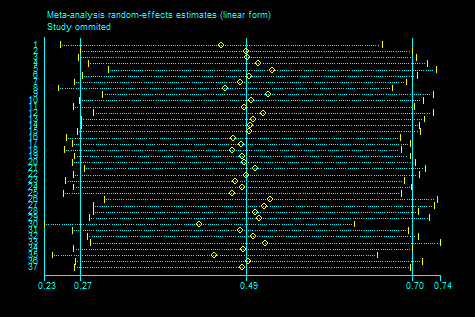


Supplementary Additional file S7. Figure8 Sensitivity analysis for rs1048943 and lung cancer risk in the recessive model


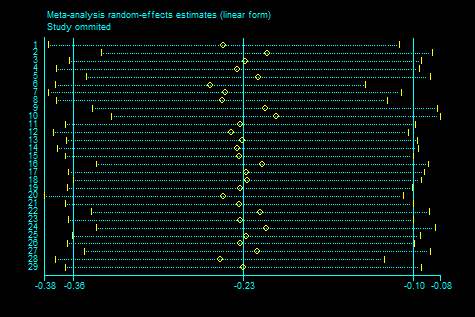


Supplementary Additional file S7. Figure9 Sensitivity analysis for rs2031920 and lung cancer risk in the heterozygote comparison model


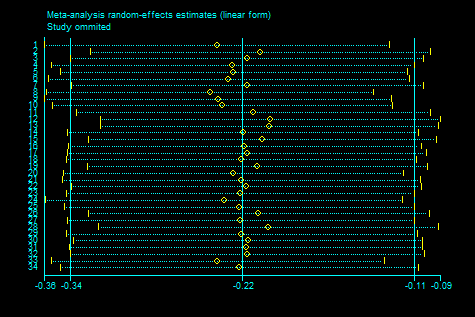


Supplementary Additional file S7. Figure10 Sensitivity analysis for rs2031920 and lung cancer risk in the dominant model


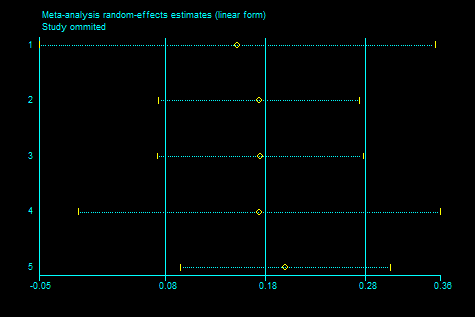


Supplementary Additional file S7. Figure11 Sensitivity analysis for rs2308321 and lung cancer risk in the heterozygote comparison model


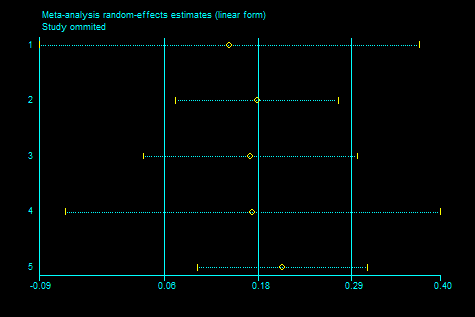


Supplementary Additional file S7. Figure12 Sensitivity analysis for rs2308321 and lung cancer risk in the dominant model


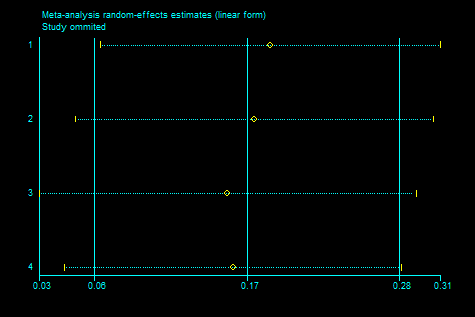


Supplementary Additional file S7. Figure13 Sensitivity analysis for rs2735383 and lung cancer risk in the dominant model


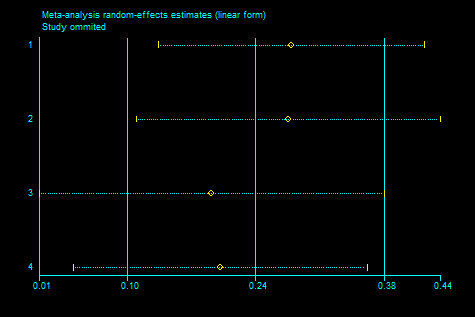


Supplementary Additional file S7. Figure14 Sensitivity analysis for rs2735383 and lung cancer risk in the recessive model


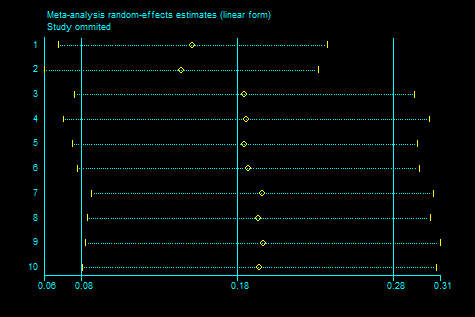


Supplementary Additional file S7. Figure15 Sensitivity analysis for rs2736098 and lung cancer risk in the heterozygote comparison model


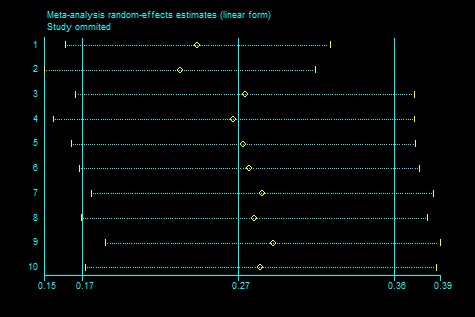


Supplementary Additional file S7. Figure16 Sensitivity analysis for rs2736098 and lung cancer risk in the dominant model


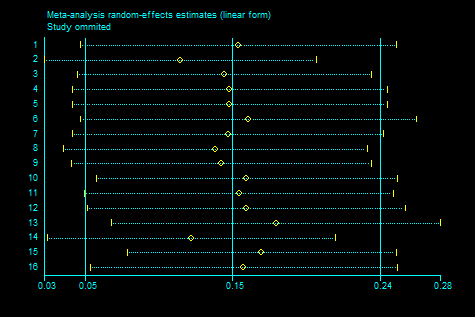


Supplementary Additional file S7. Figure17 Sensitivity analysis for rs1800975 and lung cancer risk in the recessive model


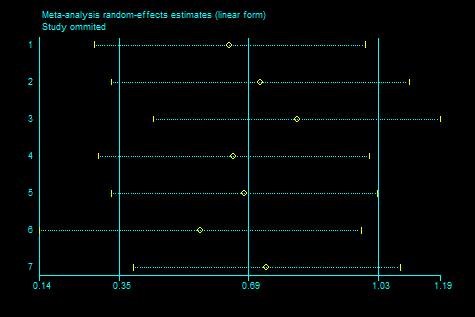


Supplementary Additional file S7. Figure18 Sensitivity analysis for rs3213245 and lung cancer risk in the homozygote comparison model


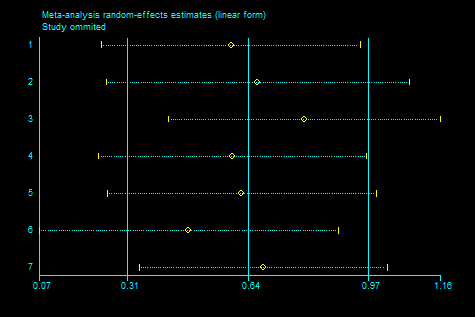


Supplementary Additional file S7. Figure19 Sensitivity analysis for rs3213245 and lung cancer risk in the recessive model


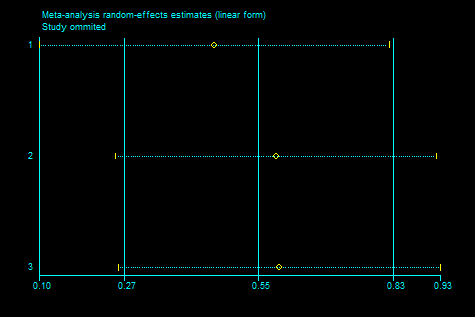


Supplementary Additional file S7. Figure20 Sensitivity analysis for rs12740674 and lung cancer risk in the homozygote comparison model


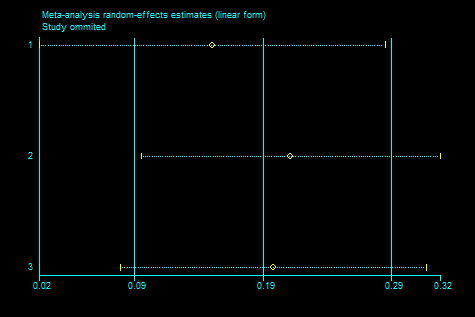


Supplementary Additional file S7. Figure21 Sensitivity analysis for rs12740674 and lung cancer risk in the dominant model


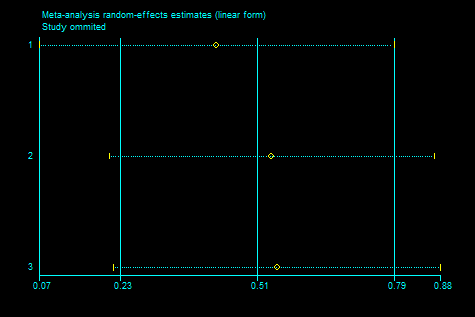


Supplementary Additional file S7. Figure22 Sensitivity analysis for rs12740674 and lung cancer risk in the recessive model
